# Elucidating the role of earth alkaline doping in perovskite-based methane dry reforming catalysts†

**DOI:** 10.1039/d1cy02044g

**Published:** 2022-01-06

**Authors:** Parastoo Delir Kheyrollahi Nezhad, Maged F. Bekheet, Nicolas Bonmassar, Albert Gili, Franz Kamutzki, Aleksander Gurlo, Andrew Doran, Sabine Schwarz, Johannes Bernardi, Sebastian Praetz, Aligholi Niaei, Ali Farzi, Simon Penner

**Affiliations:** Reactor & Catalyst Research Lab, Department of Chemical Engineering, Faculty of Chemical and Petroleum Engineering, University of Tabriz Tabriz Iran; Fachgebiet Keramische Werkstoffe/Chair of Advanced Ceramic Materials, Institut für Werkstoffwissenschaften und -technologien, Technische Universität Berlin Hardenbergstr. 40 10623 Berlin Germany; Department of Physical Chemistry, University of Innsbruck Innrain 52c A-6020 Innsbruck Austria simon.penner@uibk.ac.at +4351250758199 +4351250758003; Institut für Chemie, Technische Universität Berlin Sekretariat TC 8, Straße des 17. Juni 124 10623 Berlin Germany; Advanced Light Source, Lawrence Berkeley National Laboratory Berkeley California 94720 USA; University Service Center for Transmission Electron Microscopy, TU Wien Wiedner Hauptstrasse 8-10 A-1040 Vienna Austria; Institute of Optics and Atomic Physics, Technische Universität Berlin Hardenbergstraße 36 10623 Berlin Germany

## Abstract

To elucidate the role of earth alkaline doping in perovskite-based dry reforming of methane (DRM) catalysts, we embarked on a comparative and exemplary study of a Ni-based Sm perovskite with and without Sr doping. While the Sr-doped material appears as a structure-pure Sm_1.5_Sr_0.5_NiO_4_ Ruddlesden Popper structure, the undoped material is a NiO/monoclinic Sm_2_O_3_ composite. Hydrogen pre-reduction or direct activation in the DRM mixture in all cases yields either active Ni/Sm_2_O_3_ or Ni/Sm_2_O_3_/SrCO_3_ materials, with albeit different short-term stability and deactivation behavior. The much smaller Ni particle size after hydrogen reduction of Sm_1.5_Sr_0.5_NiO_4_, and of generally all undoped materials stabilizes the short and long-term DRM activity. Carbon dioxide reactivity manifests itself in the direct formation of SrCO_3_ in the case of Sm_1.5_Sr_0.5_NiO_4_, which is dominant at high temperatures. For Sm_1.5_Sr_0.5_NiO_4_, the CO : H_2_ ratio exceeds 1 at these temperatures, which is attributed to faster direct carbon dioxide conversion to SrCO_3_ without catalytic DRM reactivity. As no Sm_2_O_2_CO_3_ surface or bulk phase as a result of carbon dioxide activation was observed for any material – in contrast to La_2_O_2_CO_3_ – we suggest that oxy-carbonate formation plays only a minor role for DRM reactivity. Rather, we identify surface graphitic carbon as the potentially reactive intermediate. Graphitic carbon has already been shown as a crucial reaction intermediate in metal-oxide DRM catalysts and appears both for Sm_1.5_Sr_0.5_NiO_4_ and NiO/monoclinic Sm_2_O_3_ after reaction as crystalline structure. It is significantly more pronounced for the latter due to the higher amount of oxygen-deficient monoclinic Sm_2_O_3_ facilitating carbon dioxide activation. Despite the often reported beneficial role of earth alkaline dopants in DRM catalysis, we show that the situation is more complex. In our studies, the detrimental role of earth alkaline doping manifests itself in the exclusive formation of the sole stable carbonated species and a general destabilization of the Ni/monoclinic Sm_2_O_3_ interface by favoring Ni particle sintering.

## Abstract 2

To elucidate the role of earth alkaline doping in perovskite-based dry reforming of methane (DRM) catalysts, we embarked on a comparative and exemplary study of a Ni-based Sm perovskite with and without Sr doping.

## Graphical abstract

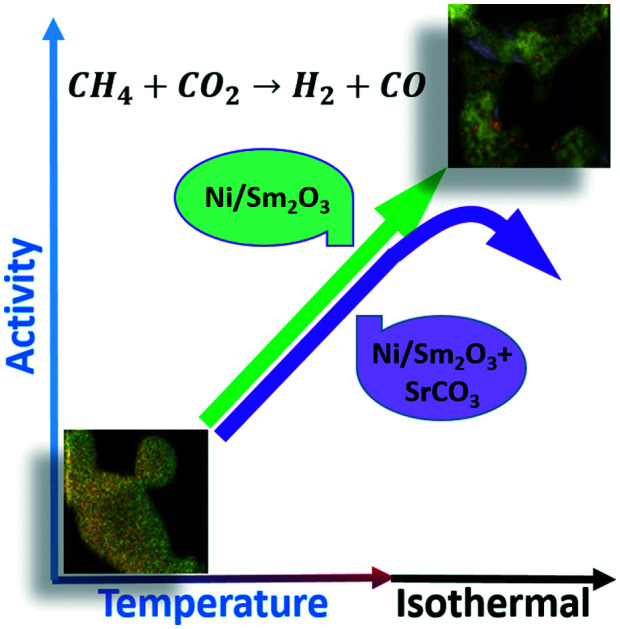

## 1. Introduction

The dry reforming of methane (DRM) reaction represents a convenient method for the simultaneous abatement of two harmful greenhouse gases carbon dioxide and methane.^1–19^ In recent years, several material classes have been screened for the development of promising catalysts and a better understanding of the reaction mechanism on an atomic level.^20–25^ Materials include intermetallic compounds, oxides, or metal-oxide systems. Mechanistic-wise, there is consensus that a bifunctional operating mechanism for both methane and carbon dioxide activation usually prevails.^26–28^ While the archetypical metal-based methane dry reforming catalyst nickel is capable of both methane and carbon dioxide activation,^29^ the serious drawbacks of Ni particle sintering at high temperatures have led to the development of more complex catalytic entities. On the most advanced material level, metal-oxide systems evolving from the *in situ* decomposition and self-activation of more complex structures during dry reforming of methane represent the state-of-the-art.^17,18,23,27,28,30,31^ Perovskite-based materials are especially well-suited to prepare metal-oxide systems with superior catalytic properties following *in situ* activation, as their ABO_3_ stoichiometry (A and B being tri- and/or bivalent metal ions) through careful selection of the A and B elements allows to trigger decomposition of the perovskite structure usually into a B/A_2_O_3_ entity.^23,27,28^ This concept is best appreciated by exemplifying the behavior of the archetypical LaNiO_3_ perovskite, whose DRM behavior has been scrutinized in every possible detail. LaNiO_3_ can be decomposed into a Ni/La_2_O_3_ system both by self-activation and a pre-reduction treatment in hydrogen. Ni crystallite sizes and carbonation tendency of La_2_O_3_ as a prerequisite of reversible carbon dioxide activation are strongly dependent on the pre-treatment and the doping level on the A- and B sites.^17,18,20,23,27,28^ Doping generally is a viable way to steer the structural decomposition into prospective catalytic materials not only by influencing the structural instability of the perovskite directly,^23,25^ but also by exploiting the surface chemical properties of the resulting oxide entity of the metal-oxide systems. The surface basicity and acidity of the oxide directly translate to the carbon dioxide activation properties.^32,33^ Basic surface sites enhance the carbon dioxide activation usually by reversible oxy-carbonate formation.^32,33^ In the case of LaNiO_3_, the corresponding Ni/La_2_O_2_CO_3_ interface is suspected to host the active sites.^23,27,28^ Even though reversible carbonate formation is imperative, also the presence of very stable spectator carbonates, *e.g.* BaCO_3_,^20^ in contact with Ni are reported to help in structurally stabilizing the active interface. One leading theme in the proper understanding of the catalytic activation of perovskite materials is the necessity to follow the activation by *in situ* characterization. It has been shown for LaNiO_3_ and a corresponding perovskite-related Ruddlesden–Popper structure La_2_NiO_4_, that before entering the final metal-oxide state, the perovskite structure goes through a sequence of transformations, including the formation of oxygen-deficient structures, transient oxide phases or polymorphic transitions. All of these intermediate steps could be directly related to changes in the catalytic activity.^20,27,28^ Apart from oxycarbonate formation, also the reactivity of surface-bound graphitic carbon was reported to play a crucial role in enhancing the DRM activity, especially on metal-oxide materials.^31^

In order to search for alternative DRM materials, Sm-containing catalysts have evolved as potentially promising. This is essentially referred to the fact that Sm_2_O_3_, like La_2_O_3_, is reported to be capable of forming a samarium oxycarbonate species^34–36^ and that the Ni/Sm_2_O_3_ interface showed superior ability in the DRM process.^37^ A particular important sub-class of Sm-based perovskite catalysts are the associated cobalt-containing materials. Evidence for the existence and use of SmCoO_3_ (and Sm_2_CoO_4_ intermediate) for efficient syngas production *via* DRM exists, and the potentially beneficial addition of earth alkaline metals to stabilize the active metal (*i.e.*, Co) species was highlighted. For the corresponding Ni-based materials, the situation is more complex from a structural viewpoint. Both the perovskite structure SmNiO_3_ and its Ruddlesden–Popper related phase Sm_2_NiO_4_ cannot be successfully synthesized structure-pure *via* a simple preparation and annealing process,^38,39^ since the ionic radius of the Sm^3+^ ion is already too small (compared to *e.g.*, La^3+^) to form a stable perovskite structure based on the Goldschmidt tolerance factor. Stabilization *via* doping is, therefore, necessary. To potentially take advantage of the surface chemical properties and eventually of structural stabilization, we opted for Sr^2+^ doping.

In the present contribution, we focus on a direct comparison of a Ni–Sm material prepared with and without additional Sr doping to assess the effect of doping on the structural stability and the catalytic DRM properties. Given the mere suspicion of the beneficial action of the added earth alkaline dopant, we are able to address the open questions how i) and if the structural stabilization works, ii) to assess the influence of the Sr doping on the oxy-carbonate formation, iii) to identify possible alternative reaction/carbon dioxide activation pathways in the presence of Sr doping and iv) to comparatively investigate the short-term stability and deactivation behavior.

The starting materials are a Ni(NiO)/Sm_2_O_3_ composite material (without Sr doping) and structure- and phase-pure Sm_1.5_Sr_0.5_NiO_4_ (with Sr doping). The resulting Sm_1.5_Sr_0.5_NiO_4_ stoichiometry represents a new class of a perovskite-related Ruddlesden–Popper perovskite, where decomposition of the perovskite and nickel exsolution under hydrogen atmosphere and self-activation under DRM conditions is possible. We show that the final fate of the decomposed Sm_1.5_Sr_0.5_NiO_4_ structure both after pre-treatment with hydrogen and after self-activation are Ni particles in a composite mixture with Sm_2_O_3_, SrO (and SrCO_3_ after self-activation). The transition into that state is structurally complex, and *via* synchrotron-based *in situ* XRD reveals several crystalline transient phases and through different Ni particle sizes as a consequence of (pre-)treatment also Ni-oxide interfaces of different extent and reactivity. We are able to establish structure–activity correlations by relating these changes to the catalytic DRM profiles and identifying similarities and differences to the state without Sr doping. Complementing the *in situ* studies, we characterize important structural points along the experiment axis also by *ex situ* and *in situ* X-ray photoelectron spectroscopy, electron microscopy (TEM) and, X-ray absorption (XANES) to correlate surface and bulk structural and morphological properties.

## 2. Experimental

### 2.1. Materials synthesis

Ni(O)/Sm_2_O_3_ and Sm_1.5_Sr_0_._5_NiO_4_ were synthesized using an auto-ignition sol–gel method. Stoichiometric amounts of the required amounts of Sm(NO_3_)_3_·6H_2_O, Sr(NO_3_)_2_, and Ni(NO_3_)_2_·6H_2_O were weighted and dissolved in de-ionized water. Afterwards, glycine was added to the solution in a molar ratio of NO_3_^−^ : NH_2_^−^ = 1 : 1 in order to induce the formation of glycine–metal complexes. The resulting solution was stirred at 80 °C for 2 h to evaporate excess water. The final gel-like residue was heated to 250 °C, causing self-ignition at 1200 °C and the formation of stoichiometric amounts of metal oxides. The formed powders were ground slightly in an agate mortar and finally calcined at 900 °C for 12 h. The resulting Sm_1.5_Sr_0.5_NiO_4_ and Ni(O)/Sm_2_O_3_ samples were distributed in four batches with each 50 mg to perform the catalytic treatments in different conditions namely under H_2_ (only hydrogen pre-reduction), DRM (only self-activation), H_2_-DRM (consecutive hydrogen reduction followed by DRM reaction) and DRM-DRM (two consecutive DRM cycles without hydrogen pre-reduction).

### 2.2. Structural, morphological and elemental characterization

The specific surface area prior to catalysis was assessed by BET surface quantification *via* nitrogen adsorption at 77 K. A Quantachrome Nova2000 surface and pore size analyzer was used for all measurements, yielding low BET areas of around 2 m^2^ g^−1^.

*Ex situ* powder X-ray diffraction (PXRD) was carried out using a STOE Stadi P powder diffractometer operating in transmission geometry and exploiting monochromatized MoK_α1_ radiation (*λ* = 0.7093 Å). A 2*θ* range of 64° and a step size of 0.015° was used. A focussing Ge(111) primary beam monochromator, as well as a linear position-sensitive detector system, was additionally employed.

*In situ* synchrotron-based PXRD experiments have been conducted in both DRM mixtures and under pure hydrogen at beamline 12.2.2, Advanced Light Source (ALS) at Lawrence Berkeley National Laboratory, in a cell previously described in ref. 40 and 41. All diffraction patterns were measured in angle-dispersive transmission mode with a focussed 25 keV monochromatic beam (*λ* = 0.4984 Å/30 μm spot size). The powders were heated in a 0.7 mm outer diameter quartz capillary under quasi-flowing conditions (CH_4_ : CO_2_ = 1 : 1, gas flow: 10 : 10 mL min^−1^ for DRM; pure hydrogen, gas flow 10 mL min^−1^, GSHV = 600 000 N mL h^−1^ g_cat_^−1^). Heating was performed using a SiC furnace with an infrared light source up to 800 °C at a rate of 10 °C min^−1^. All gases were injected through a 0.5 mm outer diameter tungsten tube. The patterns were recorded using a Perkin Elmer flat panel detector (XRD 1621 with dark image and strain correction.^40,41^ Rietveld refinement was performed using the FULLPROF program.^42^ The profile function 7 (Thompson-Cox-Hastings pseudo-Voigt convoluted with axial divergence asymmetry function)^43^ was used in all refinements. The resolution function of the diffractometers was obtained from the structure refinement of a LaB_6_ standard.

Surface characterization was performed using *ex situ* and *in situ* X-ray photoelectron spectroscopy (XPS). For *ex situ* studies, a Thermo Scientific MultiLab 2000 spectrometer, equipped with a monochromatic Al Kα X-ray source (*E* = 1486 eV) and an Alpha 110 hemispherical analyzer, was used. The base pressure lies in the 10^−10^ mbar range, and charging of the sample upon measurement is compensated by a flood gun supplying electrons with a kinetic energy of 6 eV. The used pass energy was 20 eV. Relevant high-resolution spectra in the relevant Sm 3d, Ni 2p, Sr 3d, C 1s, and O 1s regions have been collected and used for qualitative and quantitative analysis. For Ni, after background subtraction using a Shirley-type function, deconvolution of the Ni 2p peak into Ni^2+^ and metallic Ni components was done, obeying the spin-orbit coupling of the individual Ni 2p_1/2_ and Ni 2p_3/2_ peaks for each relevant component. The full width at half maximum has been fixed for the individual deconvoluted Ni species as an additional constraint. In contrast, the position of the Ni components was left floating to account for the constant change of the Ni chemical environment during the decomposition of Sm_1.5_Sr_0.5_NiO_4_.

The UHV system used for the *in situ* near-ambient pressure X-ray photoelectron spectroscopy (NAP XPS) is a customized SPECS setup. Maintaining a base pressure below 10^−10^ mbar, it comprises an analysis chamber, which can be backfilled with oxidative, reductive and reactive gas atmospheres up to 30 mbar, a PHIOBOS 150 NAP hemispherical energy analyzer with an 1D-DLD detector, a μFOCUS 600 NAP monochromatic small spot X-ray source (Al Kα) and a Flood Gun (FG22/35). A complementary mass spectrometer allows the operando surface characterization. A four-axis manipulator adapted for laser heating and gas-phase reactions is used for all experiments. High-resolution spectra of the Ni 2p, O 1s, C 1s, Sm 3d and Sr 3d regions were collected in selected temperature steps from room temperature to 750 °C. The spectra were fitted with a Shirley-type background. Quantitative analysis was based on the relative sensitivity factors (RSFs), as well as the different inelastic mean free paths. To monitor the appearance of surface-bound Ni and the carbon dioxide activation capabilities, the sample was sequentially heated in 0.2 mbar hydrogen and 0.2 mbar carbon dioxide in 50–100 °C steps from 25 °C to 800 °C, respectively.

Structural and morphological characterization has been further performed by *ex situ* transmission electron microscopy (TEM) at the University Service Facility for Transmission Electron Microscopy (USTEM) at TU Vienna using a FEI Tecnai F20 S-TWIN analytical high-resolution microscope operated at 200 kV in combination with a windowless Apollo XLTW silicon drift detector for EDX experiments.

X-ray absorption measurements were carried out with a novel self-developed wavelength-dispersive spectrometer in von Hámos geometry.^44,45^ The spectrometer is equipped with a microfocus X-ray tube, a curved highly annealed pyrolytic graphite mosaic crystal and a hybrid photon counting CMOS detector with 512 × 1030 pixel and a pixel size of 75 μm × 75 μm. The tube was operated with a voltage and current of 15.8 kV and 1870 μA for Ni K-edge, 12.8 kV and 1710 μA for Sm LIII-edge, and 19.8 kV and 14 900 μA for Sr K-edge measurements. As the Ni reference, a 2 μm Ni foil was used. All other references and samples were in powder form and prepared as wax-pellets (mixed with Hoechst Wax C), due to their low concentration of Ni. The blending of the sample and reference sample with Hoechst Wax was necessary to achieve pellets with an adequate thickness for stability. After stirring wax and sample material in a mortar to get a homogenous mixture, a pellet with a 13 mm diameter was pressed by using a hydraulic pellet press with force up to 6 tons for not longer than 60 s. As the samples were measured in transmission mode, the absorption spectrum was acquired by measuring once with and once without the sample. The measurement time for each sample varied between 8 to 10 h depending on the thickness of the prepared sample. All references and samples were constantly moved during the measurements to minimize the effects of local thickness inhomogeneity. The beam size on the samples is around 3 mm × 3 mm. The gathered spectral range is covering the Ni K absorption edge at 8332 eV. Normalization of the spectra, as well as linear combination fitting (LCF), was done by using the XAS analyzing and processing software ATHENA which is part of the Demeter software package.^46^

### 2.3. Catalytic testing

The performance of the catalysts with respect to DRM activity was tested in a home-built quartz fixed-bed tubular flow reactor (inner diameter = 7 mm, outer diameter = 9 mm, catalyst bed length: 2.5 cm). Each gas required for DRM (CH_4_ : CO_2_ : He = 1 : 1 : 3 ratio; GHSV = 60 000 N mL g_cat_ h^−1^) was injected through a corresponding mass flow controller (MKS), where helium acts both as a carrier gas and as a heat conductor. 100 mg catalyst powder was used for each test and homogeneously distributed using thoroughly degassed quartz wool over the entire catalyst bed. Heating was achieved using a Linn High Therm furnace up to 800 °C at a fixed rate of 10 °C min^−1^. An S-type Pt/PtRh thermocouple placed in close vicinity to the sample ensured accurate temperature reading. The output gas was continuously extracted through a capillary and analyzed by a quadrupole mass spectrometer mounted in cross-beam geometry (Balzers QMA 125). Hydrogen pre-treatments were carried out in 1 bar flowing hydrogen at a rate of 10 mL min^−1^ up to 800 °C. We have performed several DRM cycles after the selected experimental sequences (*cf.* section 2.1.). In this respect, the term “first” or “second” cycle is used for DRM measurements, which have been consecutively performed, irrespective of the pre-treatment.

Due to the bifunctional operating mechanism of DRM catalysts on perovskite basis, normalization of the catalytic activity solely to the surface area of the exsolved Ni particles would grossly overestimate the intrinsic, structure-insensitive catalytic role of Ni and is therefore not considered (*i.e.*, calculating metal surface-based turnover frequencies is not meaningful during *in situ* activation). To derive a qualitative understanding and judgment of structure–activity correlations from temperature-programmed DRM experiments, we rely on onset temperatures of catalytic activity, conversion (%) and H_2_/CO product ratios. The relative catalytic activity is, therefore, compared on the basis of conversion *vs.* temperature plots.

## 3. Results and discussion

### 3.1. Catalyst with Sr doping: the role of Sm_1.5_Sr_0.5_NiO_4_ in DRM

#### Stability and decomposition of Sm_1.5_Sr_0.5_NiO_4_ after selected pre-treatments in hydrogen and after activation in the DRM mixture: benchmark states

**Fig. 1:**
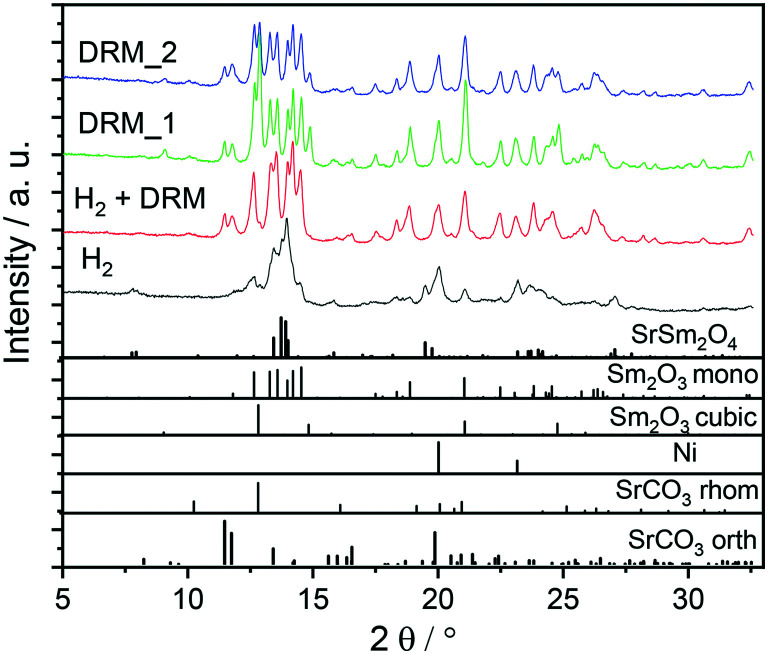
*Ex situ* XRD patterns of Sm_1.5_Sr_0.5_NiO_4_ after selected pre-treatments and activation experiments. Black trace: hydrogen treatment under flowing pure hydrogen, red trace: post-DRM treatment after pre-reduction in hydrogen, green trace: DRM treatment without hydrogen pre-treatment, blue trace: after two consecutive DRM cycles. Final temperature: 800 °C, heating ramp: 10 °C min^−1^. The lower panel indicates the phase assignment to the respective reference structures. The unindexed broad shoulder at 2*θ* ~ 12.05° corresponds to (002) reflection of graphitic carbon. Wavelength: *λ* = 0.7093 Å.

To evaluate the general stability of the Sm_1.5_Sr_0.5_NiO_4_ perovskite structure under reduction and DRM conditions, we evaluated the bulk structure first by both *ex situ* XRD and TEM. The initial X-ray diffractogram in Fig. 1 reveals the Sm_1.5_Sr_0.5_NiO_4_ structure with a minor amount of cubic and monoclinic Sm_2_O_3_. Decomposition into a Ni/Sm_2_O_3_/SrO composite is observed upon treatment in either hydrogen or a carbon dioxide/methane mixture at 800 °C under flowing conditions (pure hydrogen or carbon dioxide : methane = 1 : 1 mixture), with the simultaneous presence of both orthorhombic and rhombohedral SrCO_3_ after DRM operation. Carbonation of SrO can also be observed after a post-DRM treatment after a hydrogen pre-treatment. The resulting diffractograms are almost identical (green *vs.* red diffractogram in Fig. 1). A stationary phase composition after multiple consecutive DRM cycles is observed (blue diffractogram), which exhibits only minor differences to the post hydrogen-DRM measurement. The phase compositions of all samples are quantified using Rietveld refinement of XRD data (Table 1). Small amounts of graphitic carbon are observed in the spent catalyst after the DRM treatment, before disappearing during the short-term stability tests for 12 hours (Fig. S1 and S2†). The presence of graphitic carbon is confirmed by using two models for the Rietveld refinement analysis (*i.e.*, with and without including graphitic carbon). As shown in Fig. S1,† the inclusion of graphitic carbon in the analysis leads to a better match between experimental and calculated diffraction data, in particular for the peak at 2*θ* ∼ 12.05° corresponding to (002) reflection of graphitic carbon. As shown in Table 1, the decomposition of the Sm_1.5_Sr_0.5_NiO_4_ catalyst in H_2_ results in a smaller crystallite size of Ni compared to decomposition under DRM conditions. Moreover, a higher amount of c-Sm_2_O_3_ is formed by decomposing the catalyst under DRM, while a negligible amount of this phase (∼2.4 wt%) is formed in the H_2_ atmosphere.

**Table 1:** Quantitative analysis of phase compositions and crystallite size of Ni in the spent catalysts determined by Rietveld refinement analysis of *ex situ* XRD data

Catalytic conditions	Sm_1.5_Sr_0.5_NiO_4_ catalyst		Ni/Sm_2_O_3_ catalyst	
	Phase composition (wt%)	Ni crystallite size (nm)	Phase composition (wt%)	Ni crystallite size (nm)
Before catalytic experiment	98.3% Sm_1.5_Sr_0.5_NiO_4_	—	68.2% m-Sm_2_O_3_	—
	1.7% m-Sm_2_O_3_		13.6% c-Sm_2_O_3_	
			18.2% NiO	
Reduction in H_2_	37.7% SrSm_2_O_4_	9.2(1)	—	—
	39.8% m-Sm_2_O_3_			
	2.4% c-Sm_2_O_3_			
	20.1% Ni			
After 1 DRM cycle	45.8% m-Sm_2_O_3_	16.0(1)	70.6% m-Sm_2_O_3_	21(1)
	22.8% c-Sm_2_O_3_		14.5% c-Sm_2_O_3_	
	15.0% o-SrCO_3_		14.9% Ni	
	1.9% rh-SrCO_3_			
	14.5% Ni			
After pre-reduction in H_2_ and 1 DRM cycle	63.9% m-Sm_2_O_3_	12.9(1)	53.2% m-Sm_2_O_3_	29.4(1)
	17.1% o-SrCO_3_		13.3% c-Sm_2_O_3_	
	1.6% rh-SrCO_3_		11.6% Ni	
	14.1% Ni		21.9% C	
	3.3% C			
After 2 consecutive DRM cycles without H_2_ pre-reduction	39.4% m-Sm_2_O_3_	19.8(1)	—	—
	10.6% c-Sm_2_O_3_			
	14.3% o-SrCO_3_			
	1.0% rh-SrCO_3_			
	11.1% Ni			
	23.6% C			
After 2 consecutive DRM cycles and stability test for 12 h	36.0% m-Sm_2_O_3_	29.3(1)	48.5% m-Sm_2_O_3_	29.4(1)
	31.9% c-Sm_2_O_3_		10.6% c-Sm_2_O_3_	
	17.3% o-SrCO_3_		10.3% Ni	
	14.8% Ni		30.6% C	
After pre-reduction in H_2_, 1 DRM cycle and stability test for 12 h	66.3% m-Sm_2_O_3_	16.3(1)	54.8% m-Sm_2_O_3_	29.7(1)
	18.9% o-SrCO_3_		11.1% c-Sm_2_O_3_	
	14.8% Ni		11.5% Ni	
			22.6% C	

**Fig. 2:**
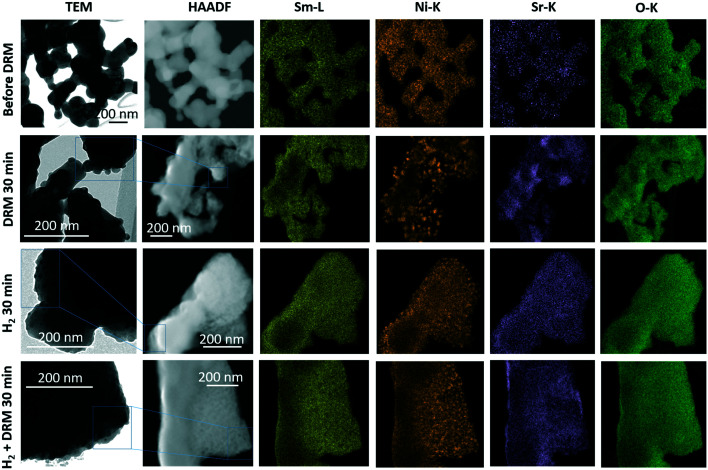
TEM/STEM overview analysis of the Sm_1.5_Sr_0.5_NiO_4_ Ruddlesden–Popper structure in the initial state (top row) and after selected *in situ* activation treatments in a 1 : 1 CO_2_ : CH_4_ dry reforming methane mixture for 30 min (second row from the top), 1 bar flowing hydrogen for 30 min (second row from the bottom) and 1 bar flowing hydrogen for 30 min followed by a treatment in a 1 : 1 CO_2_ : CH_4_ dry reforming methane mixture for 30 min (bottom-most row). Final temperatures in all cases 800 °C. The first column depicts overview bright-field TEM images, the second column the respective HAADF images alongside the EDX analysis based on the Sm-L (yellow), Ni-K (orange), Sr-K (magenta) and O-K edges (green).

The complementary structure and morphology in the initial state and after the selected pre-reduction and activation treatments by electron microscopy (Fig. 2) reveals a loose network of rounded, ∼200 nm-sized individual grains, which show a uniform elemental distribution of Sm, Ni, Sr, and O in the as-synthesized calcined state (top-most row, Fig. 2). Corroborating the XRD results, we observe the decomposition of Sm_1.5_Sr_0.5_NiO_4_ after all treatments. While the Sm and O distribution remain almost uniform, the exsolution of small Ni particles (≤30 nm in size) is prevalent both after the treatment in hydrogen and in the DRM mixture. After the latter, Sr is also increasingly randomly distributed. Most striking is the clear difference in Ni particle size after hydrogen treatment compared to after self-activation during DRM operation. Already obvious in the Ni-K EDX maps, the HRTEM images in Fig. S3† indicate a much smaller particle size after a hydrogen treatment (<15 nm, blue framed panel in Fig. S3†) in comparison to after a DRM treatment (>20 nm, red-framed panel in Fig. S3†), which agrees with the XRD results (Table 1).

Of particular relevance for DRM operation is the resistance to coking. Although the presence of especially surface carbon is not always a disadvantage and under special circumstances can act as a reactive intermediate,^31^ depositions of carbon usually lead to catalyst deactivation. To assess the coking propensity of the Sm_1.5_Sr_0.5_NiO_4_ material, Fig. S4† shows the elemental distribution before and after an *in situ* activation treatment in an 1 : 1 CO_2_ : CH_4_ dry reforming methane mixture for 30 min with a special focus on the carbon distribution. While Sm and O do not feature strong intensity differences before and after the treatment, carbon has only slightly accumulated during the treatment, but no particular agglomeration was observed. In particular, no carbon nanotube- or whisker-like features are observed. This confirms that coking is effectively suppressed during DRM operation on Sm_1.5_Sr_0.5_NiO_4_ – in strong contrast to the archetypical Ruddlesden–Popper structure La_2_NiO_4_, where coking is a particular issue.^18,20^ Although XRD analysis reveals the formation of small amounts of graphitic carbon in the spent catalyst after treatment under DRM with subsequent decomposition in H_2_ or DRM, all these graphitic carbon disappeared after the short-term stability tests for 12 hours (see Table 1). These results indicate that the formed graphitic carbon is an active intermediate species that reacts with CO_2_ to form syngas upon a longer reaction time. The formation of active graphitic carbon is also confirmed by the *in situ* XRD data discussed in section 3.2.

#### Dynamic structural response during pre-reduction and DRM activation monitored by *in situ* powder X-ray diffraction

As we have shown for the class of La-based Ni-containing perovskite and Ruddlesden–Popper structures,^20,26,28^ assessing the bulk dynamic response during DRM operation is paramount for a full understanding of the catalytic action and the establishment of structure–property relationships. Contrary to exclusively characterizing the spent catalyst state, intermediate and transient structures all feature intrinsic catalytic properties that add up to the global catalytic picture of the catalyst system. We have also assessed the structural differences of decomposition *via* pre-reduction treatments *vs.* self-activation in the DRM mixture and the resulting consequences for DRM activity. Based on these experiments, we present the dynamic structural response of Sm_1.5_Sr_0.5_NiO_4_ during defined cycles of pre-reduction and activation. These include: i) pre-reduction in hydrogen up to 800 °C, followed by a DRM treatment up to 800 °C and ii) two consecutive DRM cycles up to 800 °C each. Special focus is put on the appearance of transient sub-stoichiometric oxide phases, ternary oxide phases that form through reaction of two structural catalyst parts, species that arise through carbonation (*i.e.*, CO_2_ activation), or carbon deposition, and characterization of the final spent catalyst state.

**Fig. 3:**
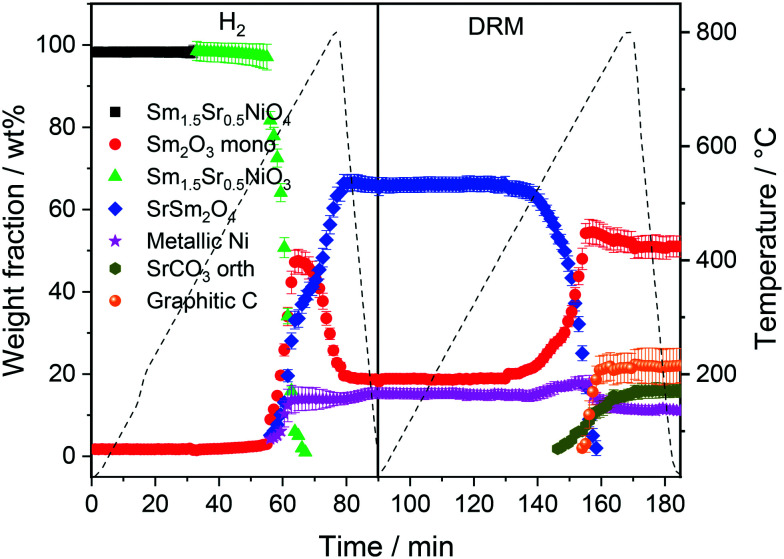
Weight fractions of different crystalline phases formed during the consecutive heating and cooling cycles of Sm_1.5_Sr_0.5_NiO_4_ in H_2_ and DRM atmospheres as a function of temperature and time obtained by Rietveld refinement of the *in situ* collected XRD patterns.

The first sequence of the hydrogen pre-treatment is shown as a set of *in situ* collected temperature-dependent X-ray diffraction patterns in Fig. S5† panels A–B and as weight-fraction analysis in Fig. 3. Sm_1.5_Sr_0.5_NiO_4_ is quantitatively transformed into oxygen-deficient Sm_1.5_Sr_0.5_NiO_3_ (which remains stable under reductive atmosphere up to 600 °C) following the reaction equationSm_1.5_Sr_0.5_NiO_4_ + H_2_ → Sm_1.5_Sr_0.5_NiO_3_ + H_2_OThe decomposition of Sm_1.5_Sr_0.5_NiO_3_ is accompanied by the simultaneous appearance of metallic Ni, monoclinic Sm_2_O_3_ and the SrSm_2_O_4_ spinel phase (space group *Cmcm*).^47^

**Fig. 4:**
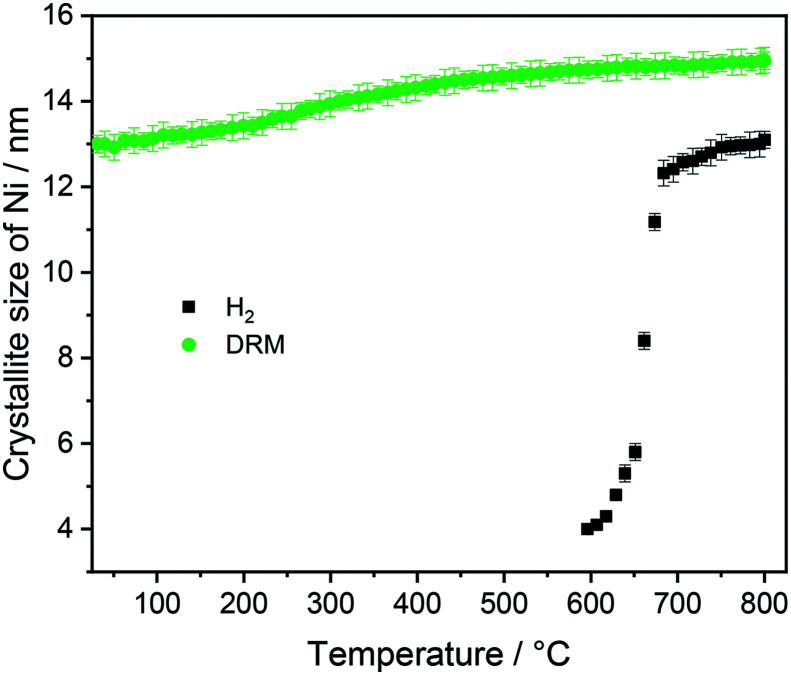
Crystallite size evolution of metallic Ni formed during heating Sm_1.5_Sr_0.5_NiO_4_ up to 800 °C in H_2_ and DRM (after H_2_ treatment) atmosphere as a function of temperature obtained by Rietveld refinement of the *in situ* collected XRD patterns.

At 700 °C, the stability limit of monoclinic Sm_2_O_3_ is reached, causing an enhancement in the formation of SrSm_2_O_4_. This transformation also proceeds during the cooling stage until a stationary composition of 65 wt% SrSm_2_O_4_, 18 wt% monoclinic Sm_2_O_3_ and 17 wt% metallic Ni is reached. Closely connected to the structural transformations is the evolution of the Ni crystallite size during hydrogen pre-reduction (Fig. 4). In the early stage of Ni exsolution starting at 600 °C, the Ni crystallite size triples from 4 nm to 12 nm within a narrow temperature window of 50 °C up to 650 °C, above which it essentially stagnates up to 800 °C. The evolution of Ni and monoclinic Sm_2_O_3_ is more or less correlated. The further accelerated formation of SrSm_2_O_4_ resulting from monoclinic Sm_2_O_3_ decomposition has only little effect on the Ni crystallite size.

In the subsequent DRM experiment (Fig. S3† panels C and D and Fig. 3), the Ni/monoclinic Sm_2_O_3_/SrSm_2_O_4_ interface is structurally stable up to 550 °C. Intertransformation of SrSm_2_O_4_ and monoclinic Sm_2_O_3_ proceeds until 700 °C, accompanied by the parallel formation of orthorhombic SrCO_3_ at and above 650 °C according to the reaction equationSrSm_2_O_4_ + CO_2_ → SrCO_3_ + Sm_2_O_3_

Graphitic carbon starts to be formed at 660 °C, suggesting the activation of the catalyst by decomposition of CH_4_. Above 700 °C, a stationary composition – also remaining unaltered during re-cooling – of 51 wt% monoclinic Sm_2_O_3_, 16 wt% orthorhombic SrCO_3_, 22 wt% graphitic carbon and 11 wt% metallic Ni is reached. The final phase composition of the recovered sample at room temperature with respect to Sr-, Sm-, and Ni-containing phases is comparable to that observed in the spent catalyst after H_2_ pre-treatment and one cycle of DRM (Table 1). A considerable higher stability of SrSm_2_O_4_ under hydrogen compared to DRM atmospheres is observed. The amount of metallic Ni remains essentially constant throughout the hydrogen pre-treatment above 700 °C, re-cooling to room temperature and the entire DRM operation. This goes along with only a minor increase in Ni crystallite size during DRM operation from 13 to 15 nm. Important for the understanding of the reaction mechanism and the establishment of structure–activity correlations, we note the apparent missing of a crystalline bulk Sm-oxycarbonate phase Sm_2_O_2_CO_3_ throughout the experiment. Such oxycarbonate phases are usually associated with the reversible CO_2_ capture/release cycle necessary for high DRM activity and are a recurrent theme for La-based perovskite catalysts.^17,18,26^ To check for the possibility of the existence of exclusively surface-bound oxy-carbonates, we performed an *in situ* X-ray photoelectron spectroscopic heating experiment in 0.2 mbar carbon dioxide between 25 °C and 750 °C (Fig. S6†) after a hydrogen pre-treatment to 800 °C to induce the formation of a Ni/Sm_2_O_3_/SrO material. The experimental conditions match those of the *in situ* XRD experiments. Corroborating the *in situ* XRD results, carbonation of Sr (presumably as SrCO_3_) is visible at 500 °C in the Sr 3d spectra. On the contrary, no indication of the formation of Sm (oxy) carbonates is observed in the respective Sm 3d spectra. In fact, neither in the bulk, nor on the surface such species are visible.

**Fig. 5:**
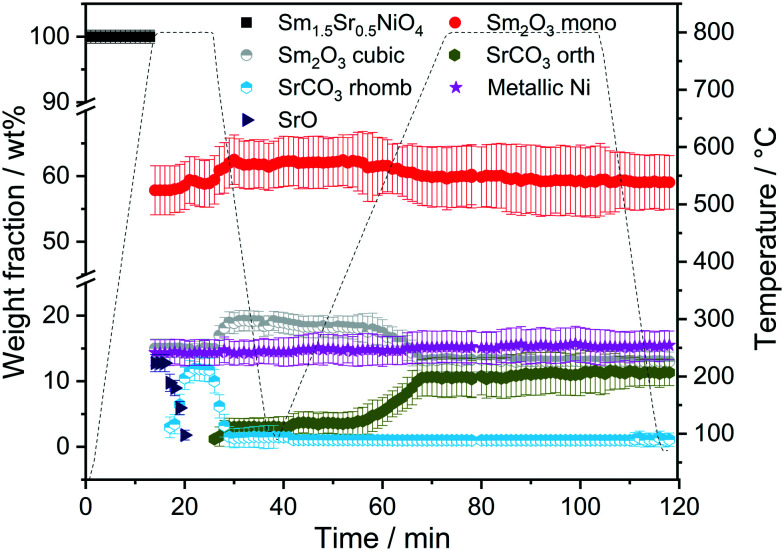
Weight fractions of different crystalline phases formed during heating and cooling Sm_1.5_Sr_0.5_NiO_4_ in DRM atmospheres (CO_2_ : CH_4_ = 1 : 1) for 10 and 30 min as a function of temperature and time obtained by Rietveld refinement of the *in situ* collected XRD patterns_._

To determine whether the structural state during and after hydrogen reduction is different from direct self-activation in the dry reforming mixture, Fig. 5 and S7† panels A–B show the corresponding analysis of the *in situ* X-ray diffraction patterns during two consecutive DRM cycles. Several differences to the hydrogen pre-treatment arise. At first, Sm_1.5_Sr_0.5_NiO_4_ is remarkably stable under DRM operation up to 800 °C. Decomposition of Sm_1.5_Sr_0.5_NiO_4_ during the isothermal period at 800 °C occurs within minutes yielding a phase mixture of metallic Ni, SrO and a mixture of cubic and monoclinic Sm_2_O_3_. Within the isothermal period, complex structural transformations involve carbonation of SrO into rhombohedral SrCO_3_ and the subsequent polymorphic intertransformation of rhombohedral SrCO_3_ and orthorhombic SrCO_3_. Upon re-cooling to room temperature and re-heating in the DRM mixture during the second cycle, no substantial structural changes occur, with the exception of the exclusive presence of orthorhombic SrCO_3_, in contrast to the first cycle where the rhombohedral modification was present. The difference between the first and second consecutive DRM cycle, which might explain this apparent existence of two different SrCO_3_ modifications, is the direct carbonation of SrO during the first cycle, whereas orthorhombic SrCO_3_ is the result of a polymorphic intertransformation. We can only speculate that the stabilization of rhombohedral SrCO_3_ during the first DRM cycle is a result of the unique structural features evolving through the decomposition of Sm_1.5_Sr_0.5_NiO_4_. It is known that orthorhombic SrCO_3_ (the stable modification under ambient conditions) undergoes a structural transition into rhombohedral SrCO_3_ upon heating, but the occurrence of rhombohedral SrCO_3_ at around 800 °C is well below the orthorhombic-to-rhombohedral transformation temperature (∼900 °C).^48–51^ The decomposition of rhombohedral SrCO_3_ upon re-cooling is in accordance with the literature, as the transformation is reversible and rhombohedral SrCO_3_ cannot be quenched to room temperature.^49^

Five points are striking: the first difference is the missing Sm_1.5_Sr_0.5_NiO_3_ and SrSm_2_O_4_ structures that were prominently observed during hydrogen pre-reduction. As Sm_1.5_Sr_0.5_NiO_3_ is apparently stabilized by oxygen vacancies, the appearance of SrSm_2_O_4_ is intrinsically connected to Sm_1.5_Sr_0.5_NiO_3._ Under the less-reducing DRM conditions, the absence of Sm_1.5_Sr_0.5_NiO_3_ and SrSm_2_O_4_ is therefore logical. Secondly, the absence of a crystalline Sm-oxycarbonate structure is equally striking. The only observed carbonated phase is again SrCO_3_. Thirdly, Fig. S8† panels A and B reveal that the full-width half-maximum of the Ni (111) reflection of the decomposed sample in the first DRM cycle is smaller than that of Ni formed in the pre-reduction in H_2_, and does not remarkably change after the second DRM cycle. Rietveld refinement reveals that the crystallite size as a result of Sm_1.5_Sr_0.5_NiO_4_ decomposition in the DRM mixture at 18 nm is much larger compared to pre-reduction in hydrogen already in the first cycle, which does not remarkably change at the end of the isothermal period at 800 °C after the second cycle. The crystallite size values of Ni determined by the *in situ* XRD experiments match those determined for Ni in the spent catalyst under the same catalytic conditions (see Table 1). Apparently, the stabilization of a smaller Ni crystallite size during hydrogen pre-reduction is a direct consequence of the transformation of Sm_1.5_Sr_0.5_NiO_4_ to Sm_1.5_Sr_0.5_NiO_3_ and further to SrSm_2_O_4_, Sm_2_O_3_ and Ni. Moreover, a recent study showed that the formation of SrCO_3_ might increase the crystallite size of metallic Ni in SrNiO_*x*_ catalysts under DRM conditions.^52^ Fourthly, although no c-Sm_2_O_3_ is formed during hydrogen pre-reduction, the decomposition of the catalyst under DRM results in 19 wt% and 13 wt% of this phase at the end of the first and second DRM cycle, respectively. These results suggest that the transition temperature between cubic and monoclinic Sm_2_O_3_ phase strongly depends on the treatment atmosphere, which agrees with previous works.^53,54^ Finally, as shown in Fig. S8C,† graphitic carbon is formed during DRM with preceding hydrogen reduction, suggesting a higher activity for the catalytic decomposition of CH_4_. However, as shown in Table 1, graphitic carbon is also observed in the spent catalyst after two consecutive DRM cycles without H_2_ pre-reduction, which might be explained by the more controlled pressure of gas mixture in the catalytic experiments as compared to the *in situ* XRD experiments. These results are consistent with previous studies highlighting that the methane and carbon dioxide conversion and the amount of carbon formation are strongly pressure-dependent.^55^

**Fig. 6:**
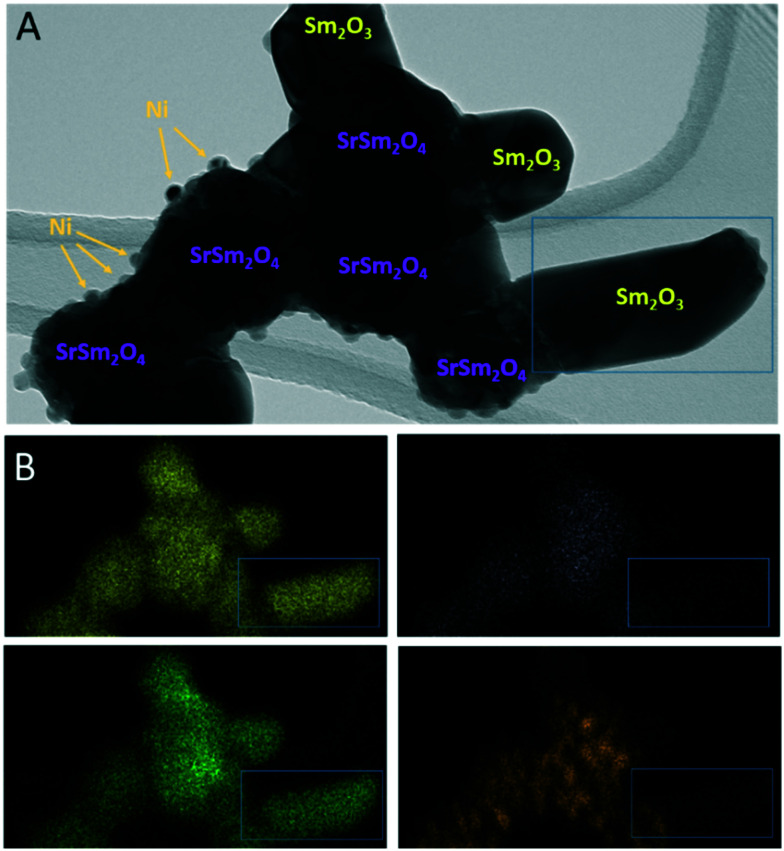
TEM/EDX analysis of Sm_1.5_Sr_0.5_NiO_4_ after hydrogen reduction at 800 °C. Panel A: Bright field overview TEM image. Phase composition of individual grains and particles as inferred from X-ray diffraction (Fig. 1) and EDX analysis is indexed. Panel B: EDX analysis using the Sm-L (yellow), Sr-K (purple), O-K (green) and Ni-K (orange) intensities.

**Fig. 7:**
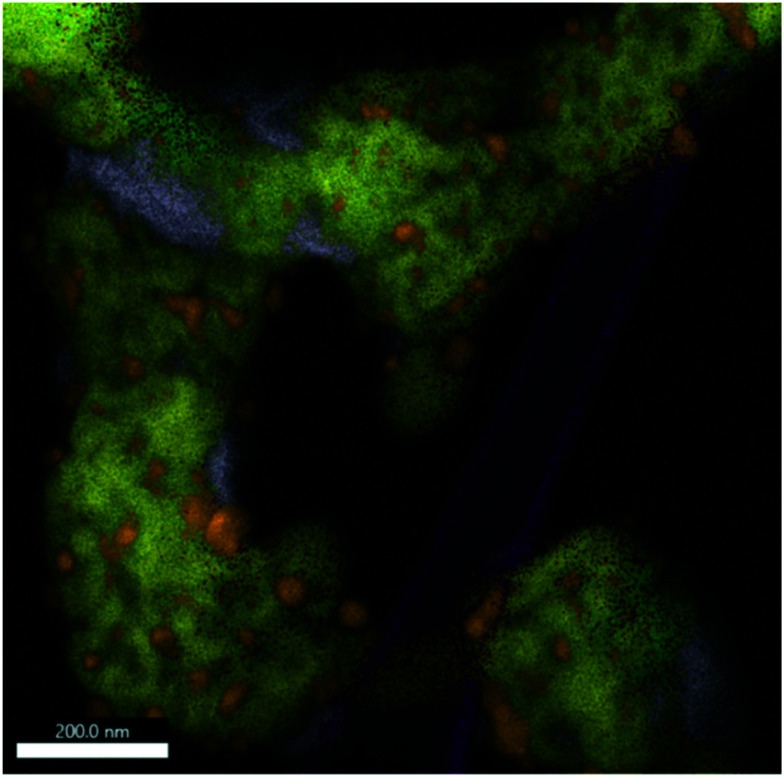
EDX analysis of Sm_1.5_Sr_0.5_NiO_4_ after a DRM reaction at 800 °C followed by an isothermal period for 30 min at 800 °C. The overlay of Sm-L (yellow), Sr-K (purple), O-K (green), C-K (magenta) and Ni-K (orange) intensities is shown. The individual maps are shown in Fig. S10.†

To corroborate the findings from *in situ* XRD and to detect eventual local structural and chemical differences after the reduction and activation treatments, Fig. 6 and 7, as well as Fig. S9 and S10 in the ESI,† highlight an in-depth electron microscopy evaluation. After hydrogen reduction at 800 °C (Fig. 6), the formation of Ni particles is observed, but exclusively locally connected to both Sm and Sr. Extra formation of Sm_2_O_3_ is also observed, but these areas are devoid of Ni particles. This is clear by comparing the intensities in the blue boxed area in the four individual EDX maps. Correlating these results to the XRD analysis, we note the exclusive presence of the SrSm_2_NiO_4_ spinel structure containing both Sm and Sr. Sm_2_O_3_, alongside Ni is also present in the composition mixture after hydrogen reduction. From the local phase analysis by TEM, we infer the crucial importance of both Sm and Sr to accelerate the exsolution of the Ni particles.

If the Sm_1.5_Sr_0.5_NiO_4_ structure is heated in the DRM mixture up to 800 °C, XRD indicated the extraordinary stability of Sm_1.5_Sr_0.5_NiO_4_ up to 800 °C and the presence of a mixture of SrO, Sm_2_O_3_ and exsolved Ni after re-cooling to room temperature (Fig. 5 and S7 and S9†). Fig. S10† shows that Ni is always found next to Sm and Sr, but never in contact with Sm alone. We still observe isolated Sm_2_O_3_ patches similar to the one in Fig. 6. In addition, isolated pure Sr-containing areas – in contrast to Sm_2_O_3_ – are also present. Carbon is mostly homogeneously distributed and also appears in areas with either pure Sm or Sr. In the lower right corner of Fig. 7 (*cf.* also Fig. S10†), an elongated Sr-containing grain is seen, with significant carbon intensity in the same local area. We conclude that this grain is associated with SrCO_3_, confirming the XRD results. XRD did not give hints towards the formation of Sm-oxy-carbonate phases (but only Sm_2_O_3_), and it appears that the carbon is mostly associated with graphitic carbon covering Sm_2_O_3_ grains.

#### Determination of the surface and bulk oxidation states by X-ray photoelectron spectroscopy and X-ray absorption studies

To characterize the surface and bulk oxidations states of the participating elements before and after the selected treatments discussed in section 3.1., we have accordingly performed *ex situ* X-ray photoelectron spectroscopy and X-ray absorption analysis.

As indicated in Fig. S11 and Table S1,† surface elemental characterization of the initial state indicate the exclusive presence of Ni (+II), as expected from the stoichiometry of Sm_1.5_Sr_0.5_NiO_4_. Pre-reduction or activation in the DRM mixture introduces a considerable amount of metallic Ni (Ni^0^), more or less independent of the treatment. On the surface, the ratio of Ni^2+^ : Ni^0^ is approximately 1 : 1, which corroborates the TEM and *in situ* XRD results.

**Fig. 8:**
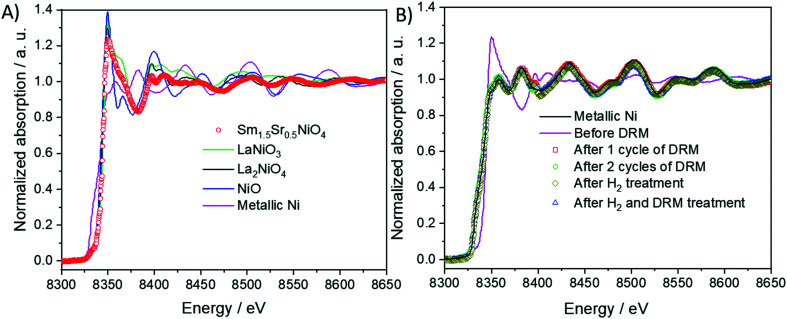
Panel A: Normalized Ni K-edge X-ray absorption near-edge structure (XANES) of Sm_1.5_Sr_0.5_NiO_4_ material compared with those of the reference materials (Ni, NiO, LaNiO_3_ and La_2_NiO_4_) and before and after different DRM experiments (panel B). The spectra are compared to that of metallic Ni as a reference material.

As shown in Fig. 8 panel A, the normalized Ni K-edge X-ray absorption near-edge structure of the initial Sm_1.5_Sr_0.5_NiO_4_ material is best fitted with the corresponding spectrum of the isostructural La_2_NiO_4_ Ruddlesden–Popper structure as the oxidation state of Ni in both compounds is +II. Accordingly, after any treatments in either hydrogen or DRM mixture, the Ni K edge structure is best fitted with metallic Ni in the oxidation state 0. As can already be deduced from the TEM experiments and the *in situ* and *ex situ* XRD analysis, the near-edge structure of the Sm LIII edge exhibits no distinct changes and is, normalized to Sm_2_O_3_, characteristic for Sm in the oxidation state +III (Fig. S12 panel A†). More distinct changes are observed for the Sr K edge (Fig. S12 panel B†). Both before and after DRM, the Sr spectrum of the initial Sm_1.5_Sr_0.5_NiO_4_ sample is essentially characterized by Sr +II, as referenced to potential SrO or SrCO_3_ phases. A chemical discrimination solely on the basis of the XANES data is difficult, as all Sr-related phases appear in the same oxidation state. It appears, however, that the fingerprints of the Sr K edge in the near-edge region of the Sm_1.5_Sr_0.5_NiO_4_ sample match those of the SrO slightly better in terms of tailing of two main peaks.

#### Catalytic DRM profiles as a function of pre-treament and self-activation

**Fig. 9:**
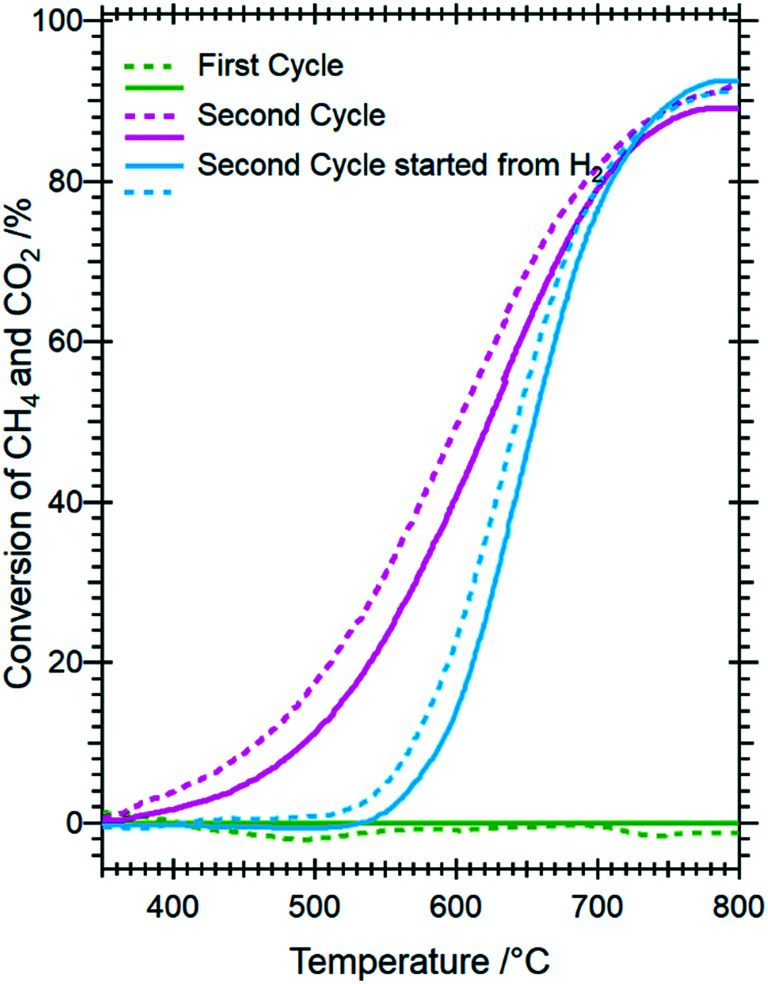
Catalytic methane dry reforming profiles of Sm_1.5_Sr_0.5_NiO_4_ as a function of hydrogen pre-treatment and activation in the DRM mixture. Reaction conditions: 1 bar flowing hydrogen; CO_2_ : CH_4_ (1 : 1) mixture. Full lines indicate methane conversion and dashed lines CO_2_ conversion.

Given the propensity for decomposition *via* pre-reduction or self-activation into a mixed Ni-oxide/carbonate composite, we tested Sm_1.5_Sr_0.5_NiO_4_ in the dry reforming of methane reaction. Fig. 9 highlights these catalytic profiles as methane/carbon dioxide conversion *versus* temperature plots with/without a pre-reduction treatment in hydrogen. To establish structure–activity correlations, we matched the experimental conditions to those of the *in situ* XRD measurements. Most striking, but understandable on the basis of the high structural stability of Sm_1.5_Sr_0.5_NiO_4_ during the first DRM cycle, Sm_1.5_Sr_0.5_NiO_4_ features hardly any DRM activity if heated in the DRM mixture in the first cycle up to 800 °C. A considerable DRM activity is only observed if either a pre-reduction treatment in hydrogen or a second consecutive DRM is conducted. Most notable is about 150 °C higher DRM onset temperature if Sm_1.5_Sr_0.5_NiO_4_ is initially decomposed *via* hydrogen reduction. The onset temperatures are 350 °C and 500 °C for the activation treatment in a DRM mixture and hydrogen, respectively. By recalling the weight fraction analysis of the *in situ* XRD patterns, it becomes clear that the onset of DRM activity is directly related to the formation of Ni/SrO-Sm_2_O_3_ followed by the appearance of orthorhombic SrCO_3_ and graphitic carbon. Performing a DRM experiment after a hydrogen pre-treatment induces carbonation of SrO roughly at 590 °C after decomposing SrSm_2_O_4_ into SrO and monoclinic Sm_2_O_3_, whereas a significant amount of orthorhombic SrCO_3_ shows up at around 400 °C to 600 °C in the second consecutive DRM cycle if no hydrogen pre-treatment is carried out. The later increase in the weight fraction of SrCO_3_ in the second DRM cycle indicates the formation of the amorphous SrO phase during the decomposition of the catalyst in the first DRM cycle. Interestingly, this activity trend is not inversely related to the Ni crystallite size, as the latter is significantly larger without pre-reduction. In fact, it appears that the activity increase and the increase in Ni crystallite size are directly related. As shown in Fig. 4 and S8A and B,† the Ni crystallite size is remarkably enlarged after DRM with subsequent H_2_ pre-reduction if compared to DRM self-activation. Although the appearance of orthorhombic SrCO_3_ is inextricably linked to accelerated methane and carbon dioxide conversion, it apparently does not stabilize smaller Ni crystallite sizes.

### 3.2. Ni/Sm_2_O_3_ without Sr doping: catalytic DRM properties and structural characterization

**Fig. 10:**
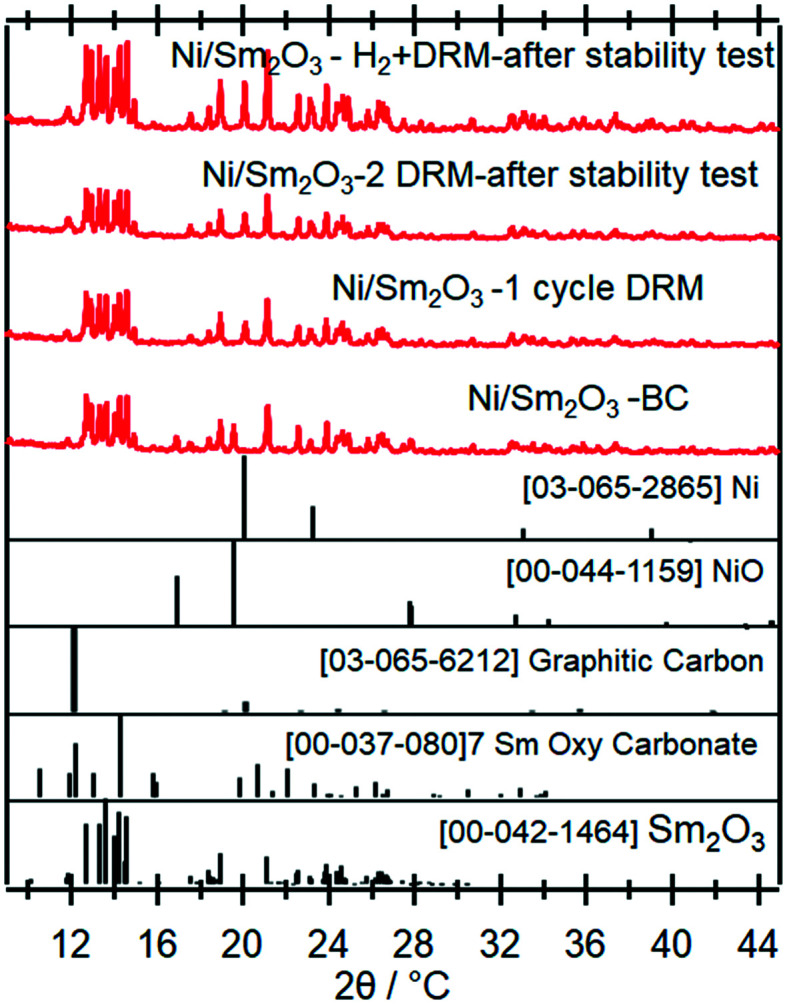
*Ex situ* X-ray diffraction patterns of the undoped pure Ni/Sm_2_O_3_ catalyst in the initial state and after selected activaton treatments as indicated. Wavelength: *λ* = 0.7093 Å.

**Fig. 11:**
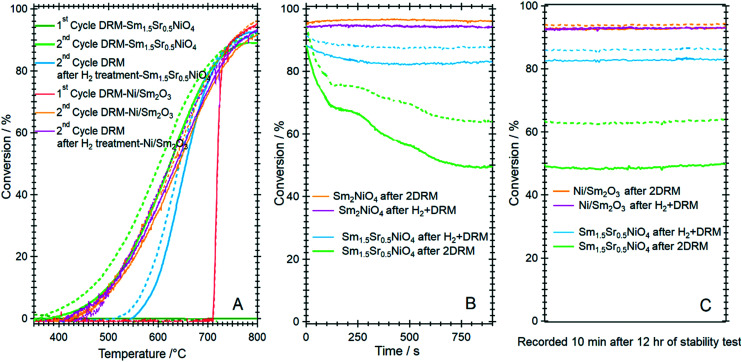
Panel A: Catalytic methane dry reforming profiles of Ni/Sm_2_O_3_ in comparison to Sm_1.5_Sr_0.5_NiO_4_ after direct activation in the DRM mixture and after hydrogen pre-activation. Panel B and C: Comparative short-term deactivation studies of Ni/Sm_2_O_3_ and Sm_1.5_Sr_0.5_NiO_4_ as a function of hydrogen pre-treatment and activation in the DRM mixture. Reaction conditions: 1 bar flowing hydrogen; CO_2_ : CH_4_ (1 : 1) mixture. Full lines indicate methane conversion and dashed lines CO_2_ conversion.

To clarify the role of Sr doping in perovskite DRM catalysts, we have synthesized the corresponding catalyst without Sr in a similar fashion. The X-ray diffraction pattern in the calcined state before activation indicates the exclusive presence of NiO (18.2 wt%), cubic Sm_2_O_3_ (13.6 wt%) and monoclinic Sm_2_O_3_ (68.2%) composite material. As expected, without Sr doping a Sm–Ni perovskite or Ruddlesden–Popper phase could structurally not be stabilized (Fig. 10). Also, the composite material is self-activating in the DRM reaction mixture, manifesting itself as a very sharp activity increase at 720 °C. In a second DRM cycle and after a corresponding hydrogen pre-activation, the catalytic DRM profiles essentially follow the other profiles with Sr doping (Fig. 11). The XRD pattern shows that the initial activity increase is clearly associated with the reduction of NiO to metallic Ni and that the active material is Ni/Sm_2_O_3_, where Sm_2_O_3_ is present in the cubic and monoclinic polymorphs.

Striking differences between the Sr-doped and undoped material arise in the short-term deactivation studies (Fig. 11, panels B and C). While the undoped Ni/Sm_2_O_3_ catalyst shows a stable DRM performance irrespective of the pre-treatment even after 12 h time-on-stream at 800 °C and also the 1 : 1 H_2_ : CO product ratio is maintained (as a consequence of catalytic DRM performance), the Sr-doped material deactivates very fast within minutes at 800 °C and the conversion – albeit stable afterwards – drops to almost 50%. Without hydrogen pre-treatment, the deactivation is most pronounced and can be directly related to the formation of a high amount of SrCO_3_ in the first DRM cycle. This in consequence induces an increase in Ni crystallite size, eventually decreases the number of accessible Ni sites and enhances the propensity towards deactivation by increasing the coverage with carbon species.^52^ As shown in Table 1, the crystallite size of Ni after DRM without hydrogen pre-reduction is ∼16 nm, which is almost 30% higher in comparison to the one with hydrogen pre-reduction (∼13 nm). Although a much larger Ni crystallite size for the catalyst without Sr doping is observed, this catalyst exhibits better short-term activity. These results suggest that the crystallite size of Ni is not the only explanation for the stable activity. Other factors, such as the presence and relative abundance of SrCO_3_, monoclinic Sm_2_O_3_ and reactive graphitic carbon phases in the catalyst, are of equal importance. As shown in Table 1 and Fig. S13 and S14,† high amounts of graphitic carbon and monoclinic Sm_2_O_3_ phases are observed in the spent Ni/Sm_2_O_3_ catalysts without Sr doping. One major issue concerns the carbon dioxide activation capability of both the Sr-doped, as well as the undoped material. For the former, we have ample evidence that the activity increase during DRM is not directly connected to the formation of a Sm_2_O_2_CO_3_ oxycarbonate phase. Neither XRD, XPS or TEM give any hint towards such a phase.

### 3.3. Discussion of the bulk and surface structural transformations and structure–activity correlation: Ni/Sm_2_O_3_*vs.* Sm_1.5_Sr_0.5_NiO_4_

To put the bulk and surface structural transformations into mechanistic and literature perspective and to subsequently correlate the structural changes to catalytic activity, we at first revisit the questions outlined in the introductory section and comparatively discuss the influence of Sr doping. Secondly, we address two peculiar structural issues that set the Sm_1.5_Sr_0.5_NiO_4_ material apart from similar catalysts: the absence of crystalline bulk Sm-oxycarbonate phases and the exclusive presence of SrCO_3_ as the only bulk-carbonated phase. As ample evidence for the generally beneficial role of adding Sm to DRM catalysts and the possibility of Sm_2_O_3_ to enter a reversible CO_2_ capture and release cycle has been presented,^57–64^ we first focus on the role of Sm_2_O_3_ in our catalysts. Despite the obvious importance of Sm_2_O_3_, the exact operating mechanism is far from clear.

The role of added earth alkaline dopants to amplify the DRM activity of perovskite and Ni-based catalysts has already been assessed in detail. The introduction of such dopants is done either through a simple impregnation step or *via* introduction into the perovskite lattice. Either way, the active component finally is an oxide phase forming a phase boundary with metals capable of methane activation, such as Ni or Co. If the dopant is introduced into the lattice, a reduction or *in situ* activation step is necessary to form this phase boundary. Several earth alkaline metal oxides such as CaO, MgO, BaO, or SrO have been used to improve the DRM activity.^21,57,64,65^ Mechanistic-wise, the beneficial action usually manifests itself either structurally by stabilizing small metal particle sizes to prevent deactivation by sintering or by directly exploiting the surface chemical properties of the oxides to enhance the CO_2_ activation capabilities of the catalyst by invoking a bifunctional synergism. Activation of CO_2_ is significantly enhanced *via* basic oxide surface sites. In the case of earth alkaline oxides, which feature the formation of very stable carbonate structures usually incapable of entering a reversible CO_2_ activation cycle, stabilization effects are reported to be more prominent. It has been shown that even the formation of stable BaCO_3_ or SrCO_3_ phases can stabilize active Ni and cobalt phases during DRM operation.^21,57,64^ Specifically, SrCO_3_ is suspected to be essential for high DRM activity *via* structurally supporting active cobalt species.^64^

We have noted the principal capability of formation of a Sm_2_O_2_CO_3_ oxy-carbonate species, but none of our bulk or surface characterization tools provides evidence for such an Sm-oxycarbonate structure during DRM operation. This leaves the presence of an amorphous Sm-oxycarbonate species or other activation pathways, proceeding through the presence of active graphitic carbon and/or monoclinic Sm_2_O_3_ as an explanation. As shown in Table 1, high amounts of monoclinic Sm_2_O_3_ and graphitic carbon remain in the spent Ni/Sm_2_O_3_ catalysts after 12 hours of DRM regardless of the pre-treatment conditions (*e.g.*, in H_2_ or self-activation during DRM).

Two types of carbon could in principle be formed on the spent nickel-based catalysts under DRM: reactive-surface-type and encapsulating-type carbon. The latter is the most common reason for catalyst deactivation, while reactive surface carbon resulting from CH_4_ decomposition is a necessary pathway for syngas production.^31^ No encapsulating-type carbon has been observed in the spent catalysts by TEM. Moreover, the short-term activity of Ni/Sm_2_O_3_ is accompanied by the formation of a high amount of graphitic carbon. These results suggest that the graphitic carbon formed under DRM in this work is of reactive-surface type. Due to the fast deactivation of Sm_1.5_Sr_0.5_NiO_4_ during the second DRM cycle, this reactive-surface carbon is successfully oxidized by CO_2_. Thus, no graphitic carbon has been observed in the spent catalyst after stability tests for 12 hours. In addition, the composition of the catalyst support or the associated interfacial properties might add to this difference in the catalytic properties. It has been reported that the reaction of carbon species with CO_2_ on Ni/La_2_O_3_/Sm_2_O_3_ catalysts could take place both *via* CO_2_ adsorption on Ni and CO_2_ adsorption at the La_2_O_3_/Sm_2_O_3_ interface.^34^ Intrinsically connected to the interfacial properties is the much higher weight % ratio of monoclinic Sm_2_O_3_ to cubic Sm_2_O_3_ (>4) after 12 hours of DRM operation. As this ratio drops to ∼1.1 for the Sr-containing catalyst with the lowest conversion rates after 12 hours time-on-stream, this explains the superior catalytic performance of Ni/Sm_2_O_3_ catalyst without Sr doping. The presence of monoclinic Sm_2_O_3_ as the main phase in the catalyst after the H_2_ pre-reduction steps (Table 1) additionally suggests a high oxygen vacancy concentration in this structure. The presence of oxygen vacancies in monoclinic Sm_2_O_3_ will facilitate CO_2_ adsorption and dissociation, as reported for other DRM catalysts.^56^ Moreover, the strong interaction of CO_2_ with monoclinic Sm_2_O_3_ also generates active sites for the adsorption and the reaction of fragmented carbon-containing intermediates, such as CH_*x*_, enhancing the CO_2_/CH_4_ conversion.^34^ Thus, hydrogen pre-reduction of the Sr-containing catalyst counteracts the detrimental poperties of the presence of Sr at least to some extent by stabilizing monoclinic Sm_2_O_3_ and increasing the ratio of monoclinic Sm_2_O_3_-to-cubic Sm_2_O_3_ (Fig. 11).

## 4. Conclusions

Our results on the influence of earth alkaline doping of perovskite-based catalysts using Sr as a representative and exemplary model in methane dry reforming yield a complex picture contrasting the simple previously reported exclusive beneficial role. Although the initial structures are already different as a consequence of the general instability of many Sm-containing perovskite materials without external doping, all catalysts irrespective of doping self-activate either during hydrogen pre-reduction or direct DRM activation. While this activation process for the undoped NiO/monoclinic Sm_2_O_3_ catalysts is associated with the simple reduction of NiO to metallic Ni, the doped Sm_1.5_Sr_0.5_NiO_4_ structure goes through a sequence of structural transformations before entering the Ni/Sm_2_O_3_/SrO(SrCO_3_) state. Transient oxygen-deficient Sm_1.5_Sr_0.5_NiO_3_ and SrSm_2_O_4_ spinel structures are both observed. The role of Sr is ambivalent: while it serves as a structural stabilizing aid, it acts as a carbon dioxide sink and structurally destabilizes the specific Ni/monoclinic Sm_2_O_3_ interface *via* enhancement of Ni particle sintering, causing catalyst deactivation. In particular, we have no evidence of the presence of a Sm-oxycarbonate structure (neither on the surface nor in the bulk), previously suspected to be responsible for the reversible carbon dioxide capture/release cycle. Rather, we have identified surface graphitic carbon in connection with the particular presence of monoclinic Sm_2_O_3_ after DRM treatments on the catalysts with and without Sr doping as a possible reactive intermediate, in line with recent studies on other metal-oxide systems.

## Conflicts of interest

There are no conflicts to declare.

## Supplementary Material

CY-012-D1CY02044G-s001
