# Supplementary material for: Elucidating the role of earth alkaline doping in perovskite-based methane dry reforming catalysts
Source: Catal Sci Technol. 2022 Jan 6;12(4):1229–44. doi: 10.1039/d1cy02044g (PMC8859525; doi:10.1039/d1cy02044g)
Supplement: CY-012-D1CY02044G-s001 [file CY-012-D1CY02044G-s001.pdf]

Electronic Supplementary Material (ESI) for Catalysis Science & Technology.
This journal is © The Royal Society of Chemistry 2022

Supporting Information

Elucidating the Role of Earth Alkaline Doping in Perovskite-Based Dry Reforming of Methane Catalysts

Parastoo Delir Kheyrollahi Nezhad[1], Maged F. Bekheet[2], Nicolas Bonmassar[3], Albert Gili[4], Franz Kamutzki[2], Aleksander Gurlo[2], Andrew Doran[5], Sabine Schwarz[6], Johannes Bernardi[6], Sebastian Praetz,[7] Aligholi Niaei[1], Ali Farzi[1] and Simon Penner[3,*]

[1]*Reactor & Catalyst Research Lab, Department of Chemical Engineering, Faculty of Chemical and Petroleum Engineering, University of Tabriz, Tabriz, Iran*

[2]*Fachgebiet Keramische Werkstoffe/Chair of Advanced Ceramic Materials, Institut für Werkstoffwissenschaften und -technologien, Technische Universität Berlin, Hardenbergstr. 40, 10623 Berlin, Germany*

[3]*Department of Physical Chemistry, University of Innsbruck, Innrain 52c, A-6020 Innsbruck, Austria*

[4] *Institut für Chemie, Technische Universität Berlin, Sekretariat TC 8, Straße des 17. Juni 124, 10623 Berlin, Germany*

[5]*Advanced Light Source, Lawrence Berkeley National Laboratory Berkeley, California 94720, USA*

[6]*University Service Center for Transmission Electron Microscopy, TU Wien, Wiedner Hauptstrasse 8-10, A-1040 Vienna, Austria*

[7] *Institute of Optics and Atomic Physics, Technische Universität Berlin, Hardenbergstraße 36, 10623 Berlin, Germany*

*Corresponding Author: simon.penner@uibk.ac.at, Tel: 004351250758003, Fax: 004351250758199

Keywords: Carbon dioxide activation, Perovskite, In situ decomposition, Nickel, Strontium carbonate

Section A: Detailed XRD patterns

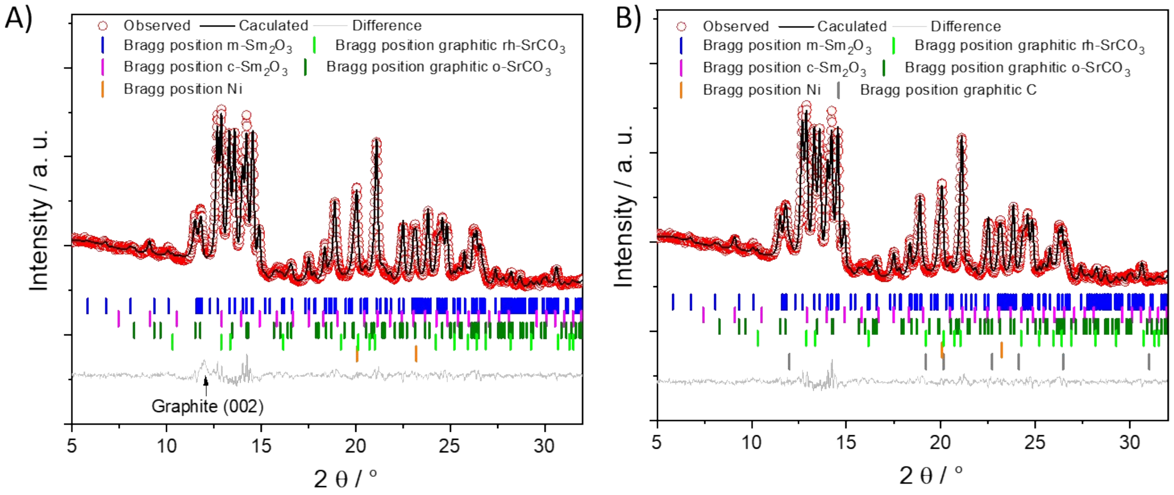

Figure S1: Structure refinement of X-ray powder diffraction data collected at room temperature for $Sm_{1.5}Sr_{0.5}NiO_4$ Ruddlesden-Popper phase after 2 consecutive DRM cycles without (A) and with (B) the inclusion of graphitic carbon in the refinement. The observed (red circle), calculated (solid black line) and difference (solid gray line) intensities, as well as the calculated Bragg reflections (tick marks) of different crystalline phases. Wavelength: λ=0.7093 Å.

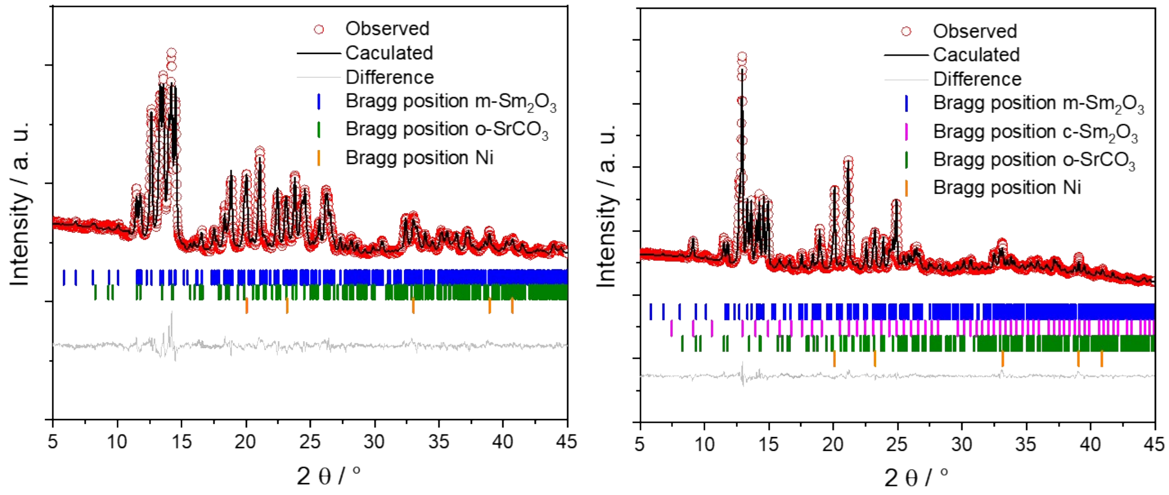

Figure S2: Structure refinement of X-ray powder diffraction data collected at room temperature for $Sm_{1.5}Sr_{0.5}NiO_4$ Ruddlesden-Popper phase after pre-reduction in H_2 and 1 DRM cycle (A) as well as after 2 consecutive DRM cycles (B), which are followed by stability tests for 12 hours. The observed (red circle), calculated (solid black line) and difference (solid gray line)

intensities, as well as the calculated Bragg reflections (tick marks) of different crystalline phases. Wavelength: λ=0.7093 Å.

Section B: HRTEM images of $Sm_{1.5}Sr_{0.5}NiO_4$ Ruddlesden-Popper phase after selected treatments in a 1:1 CO_2:CH_4 dry reforming methane mixture

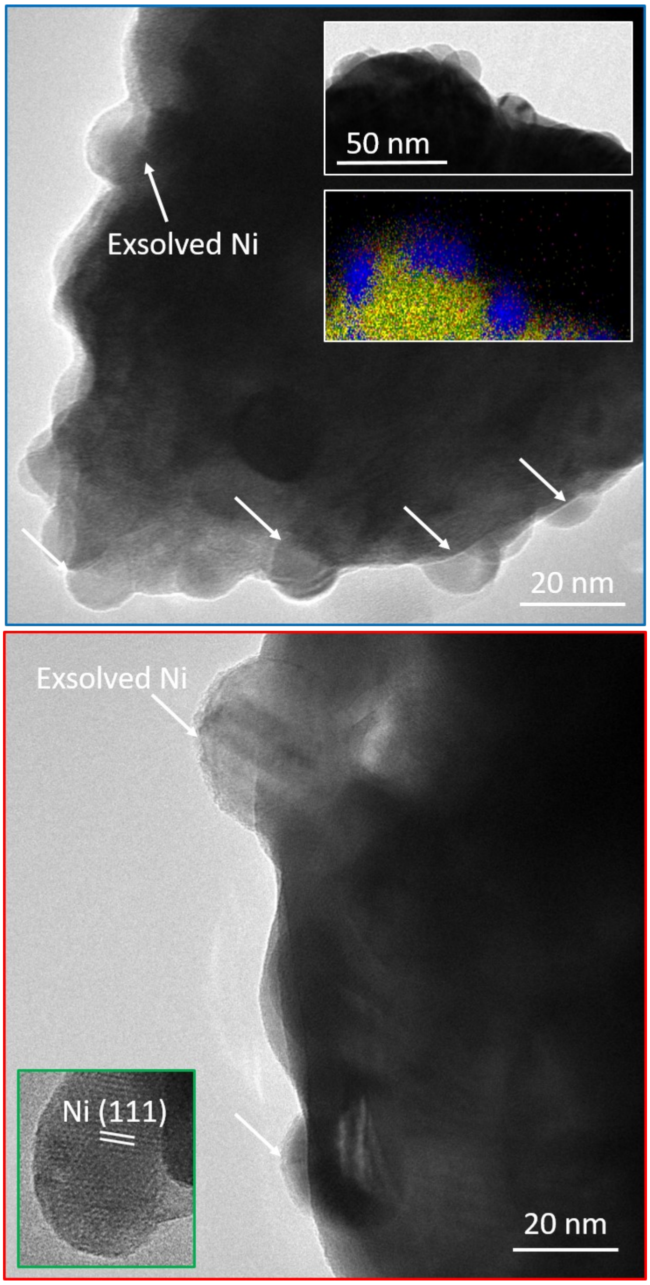

Figure S3: Detailed (HR)TEM analysis of $Sm_{1.5}Sr_{0.5}NiO_4$ Ruddlesden-Popper phase after selected treatments in a 1:1 CO_2:CH_4 dry reforming methane mixture for 30 min (bottom panel, red frame) and after 1 bar flowing hydrogen for 30 min followed by a treatment in a 1:1 CO_2:CH_4 dry reforming methane mixture for 30 min (top panel, blue frame). Exsolved Ni as small particles decorating the rims of the much larger oxide matrix are seen in both images and have been marked by white arrrows. A representative high-resolution image of a metallic Ni particle exhibiting atomically resolved Ni(111) lattice fringes is shown as inset, framed in green. The inset in the right panel highlights a comparison of a BF image (top) and an overlay of EDX intensities of Sm-L (beige), Ni-K (blue), Sr-K (lilac), C-K (red) and O-K edges (green).

Section C: Coking propensity - TEM

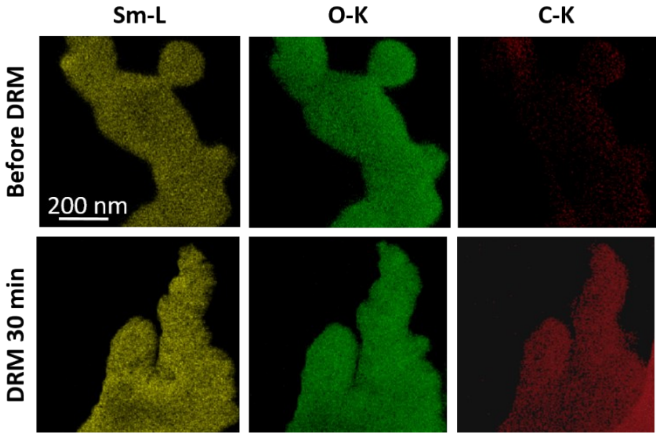

Figure S4: EDX comparison of the $Sm_{1.5}Sr_{0.5}NiO_4$ Ruddlesden-Popper phase in the initial state (top row) and after an in situ activation treatment in an 1:1 CO_2:CH_4 dry reforming of methane mixture for 30 min (bottom row). The Sm-L (beige), O-K (green) and C-K edges (red) are displayed.

Section D: In situ XRD patterns during H_2-DRM

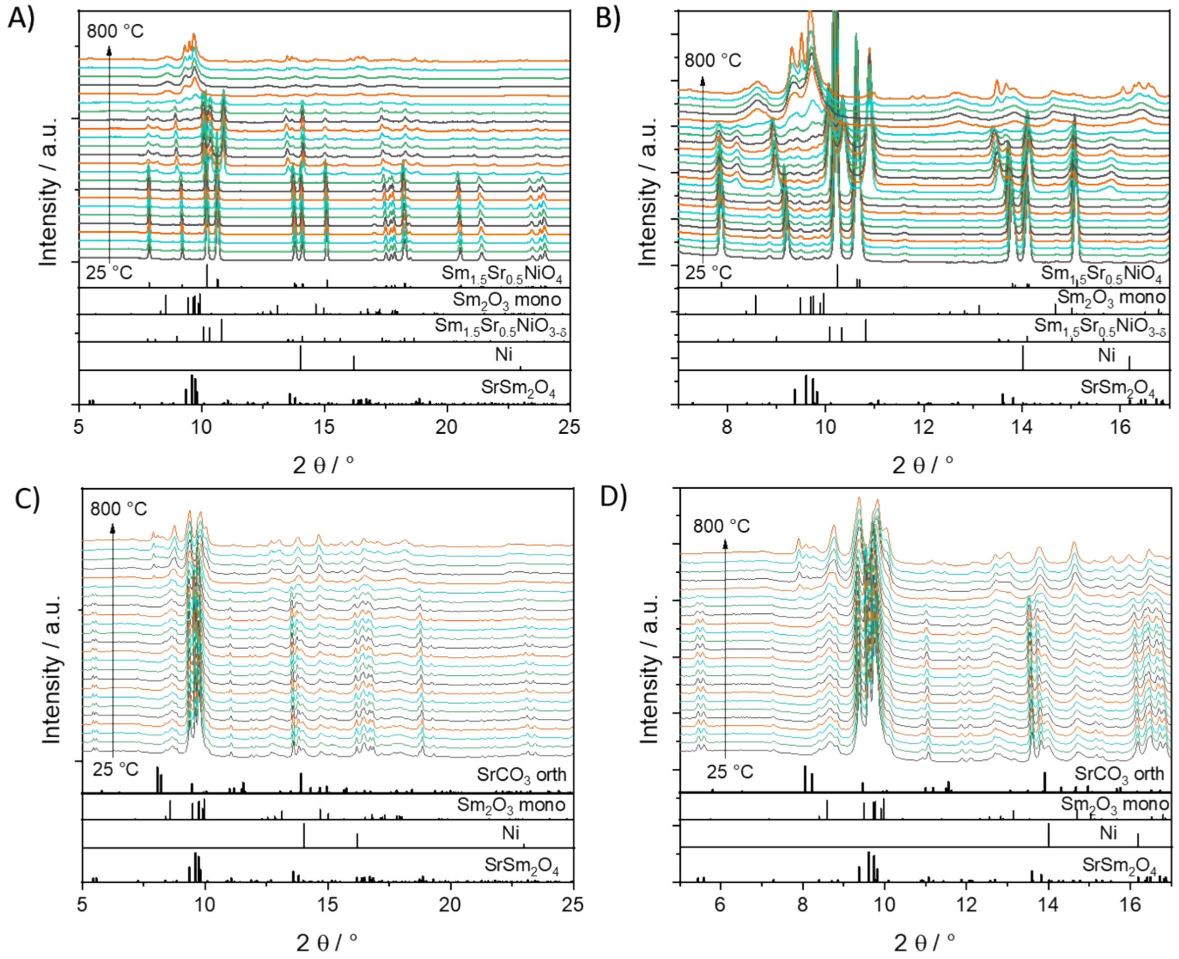

Figure S5: In situ collected XRD patterns of $Sm_{1.5}Sr_{0.5}NiO_4$ during heating up to 800 °C in H_2 atmosphere (Panels A and B) and during re-heating in the DRM mixture (Panels C and D). Panels B and D focus on a narrower 2θ window for closer analysis. The lower panel indicates the assignment to the respective reference structures. The unindexed broad shoulder at $2\theta \sim 8.48°$ corresponding to (002) reflection of graphitic carbon. Wavelength: λ=0.4984 Å.

Section E: In situ X-ray photoelectron spectroscopy

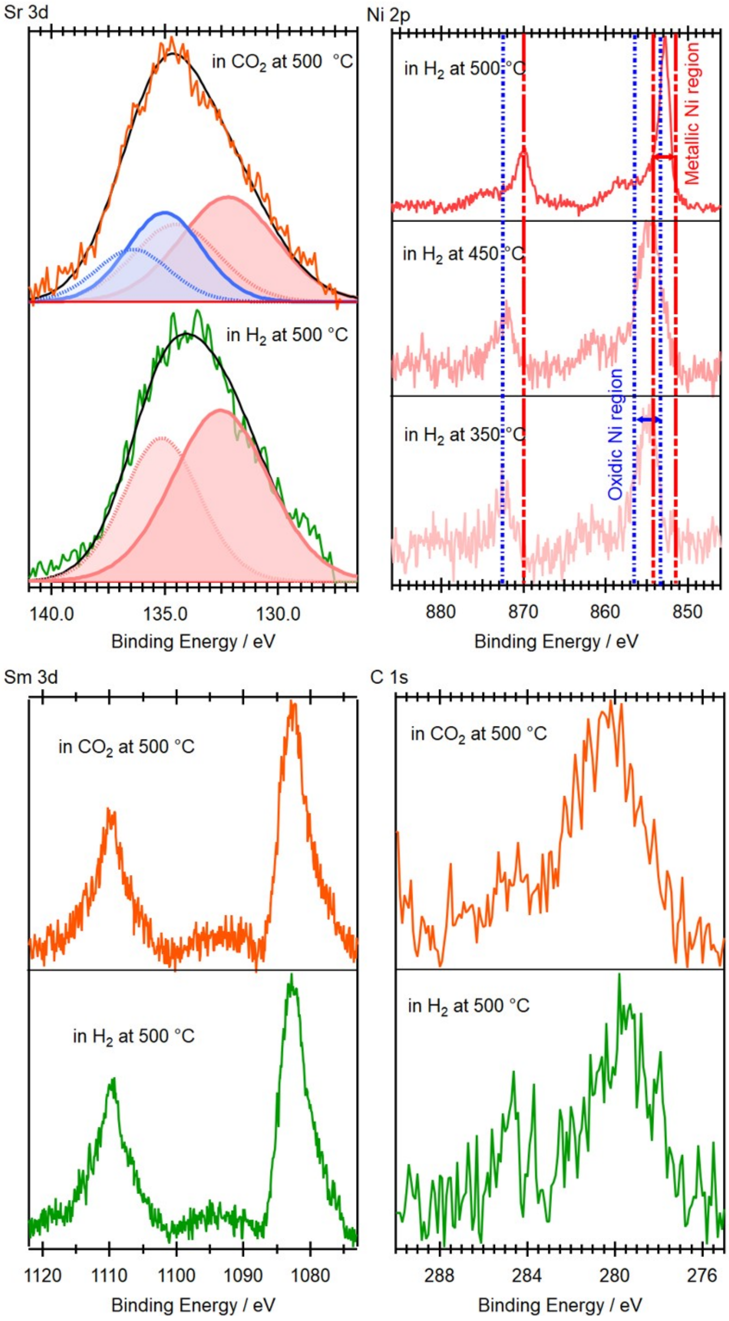

Figure S6: In situ X-ray photoelectron spectroscopy experiments on the Ni exolution and carbonation behavior of the $Sm_{1.5}Sr_{0.5}NiO_4$ material. Upper right panel: Evolution of the Ni 2p peak as a function of the hydrogen pre-treatment conditions. Upper left panel and bottom row: Direct comparison of the Sr 3d, Sm 3d and C 1s spectra collected at 500 °C in 0.2 mbar hydrogen and 0.2 mbar carbon dioxide. While the Sm 3d show no difference, the broader Sr 3d peak indicates the presence of a $SrCO_3$ phase as a consequence of reaction with carbon dioxide.

Section F: In situ XRD patterns during DRM

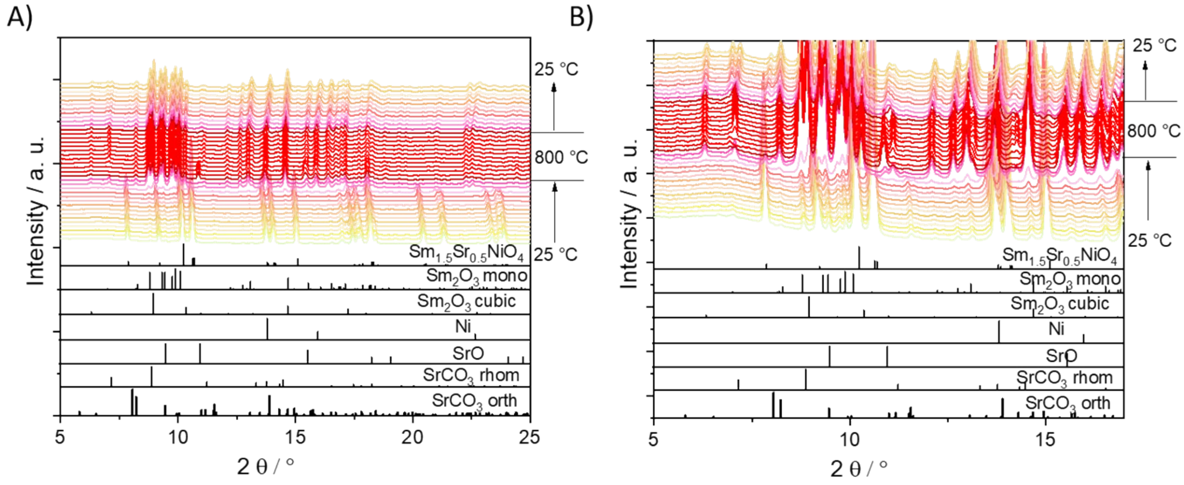

Figure S7: In situ collected XRD patterns of $Sm_{1.5}Sr_{0.5}NiO_4$ during heating up to 800 °C in DRM atmosphere for 10 min before cooling to room temperature. Panels B focus on a narrower 2θ window for closer analysis. The lower panel indicates the assignment to the respective reference structures. Wavelength: λ=0.4984 Å.

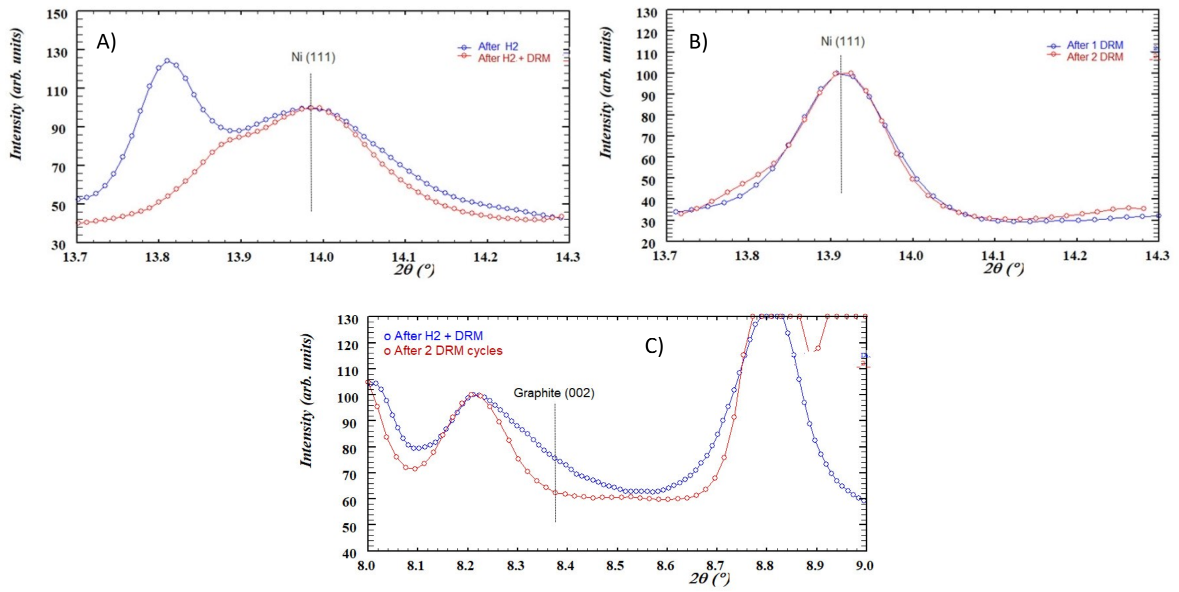

Figure S8: Comparison between the Ni (111) reflections in the collected XRD patterns of $Sm_{1.5}Sr_{0.5}NiO_4$ after cooling to room temperature from pre-reduction in H_2 and 1 DRM cycle (A), and from two consecutive cycles of DRM (B). Panel C shows the difference between the

collected XRD pattern of the recovered sample after two experiments. Wavelength: λ=0.7093 Å.

Section G: TEM characterization after one DRM cycle

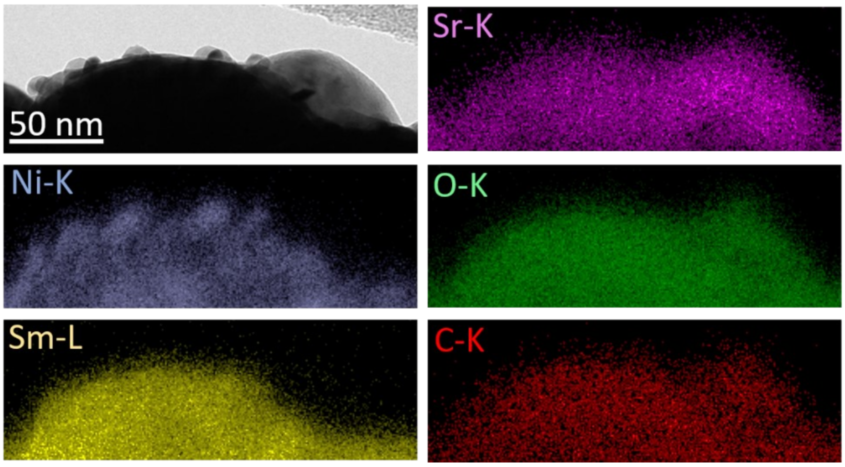

Figure S9: TEM/EDX analysis of the $Sm_{1.5}Sr_{0.5}NiO_4$ Ruddlesden-Popper phase after an in situ activation treatment in an 1:1 CO_2:CH_4 dry reforming methane mixture for 30 min (bottom row). Top left: Bright-field overview image. The Sm-L (yellow), O-K (green) and C-K edges (red), Sr-K (magenta) and Ni-K (blue) edge intensities are displayed.

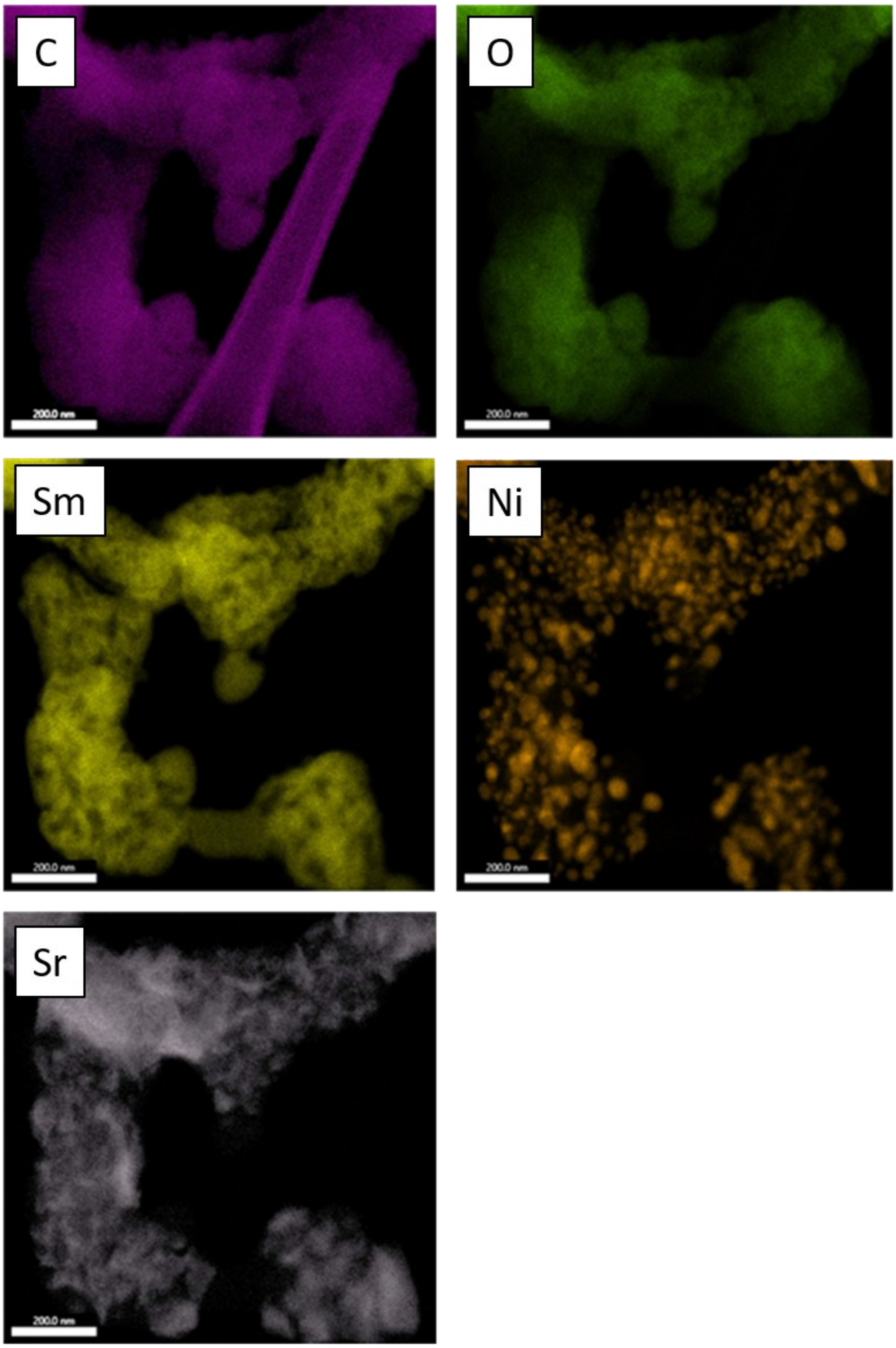

Figure S10: EDX analysis of $Sm_{1.5}Sr_{0.5}NiO_4$ after a DRM reaction at 800 °C followed by an isothermal period for 30 min at 800 °C. Sm-L (yellow), Sr-K (purple), O-K (green), C-K (pink) and Ni-K (orange) intensities are shown. Overlay shown in Figure 7.

Section H: **X-ray photoelectron spectra collected after selected pre-treatments**

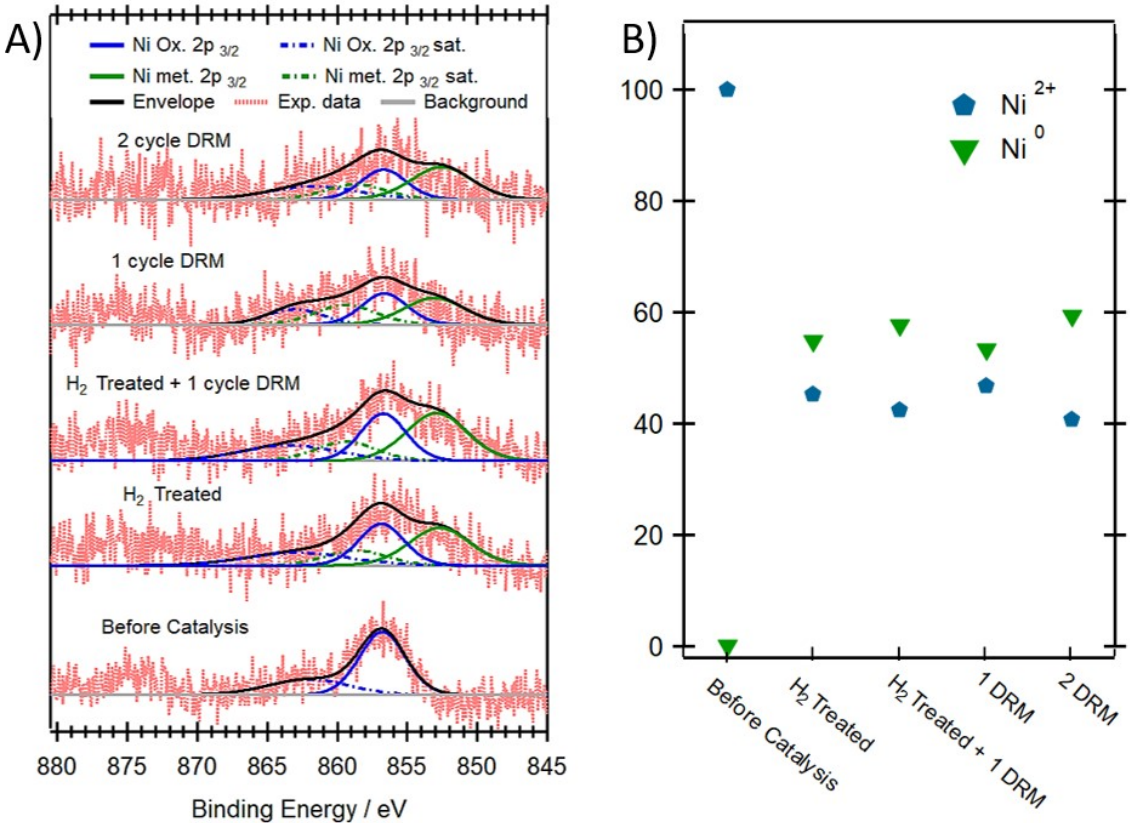

Figure S11: Panel A: High-resolution X-ray photoelectron spectra of the Ni 2p region in the initial state and after selected pre-reduction and self-activation treatments. The Ni 2p region has been deconvoluted into individual Ni components. Panel B: Quantitative analysis (in at %) of Ni^{2+} and Ni^0 as a function of treatment.

Table S1: Quantitative analysis (in at%) of Ni^{2+} and Ni^0 as a function of treatment.

XPS fitting data (Atom %) Ni 2p region for catalysts	Ni 2p $_{3/2}$ (Ni^{2+})	Ni 2p $_{3/2}$ (Ni^0)
Before catalysis	100	0
H_2 Treated (800 °C)	45.20	54.8
H_2 Treated (800 °C) + 1 DRM (800 °C)	52.5	57.5
1st cycle DRM (800 °C)	46.7	53.3
2nd cycle DRM (800 °C)	40.7	59.3

Section I: X-ray absorption spectra of the normalized Sm LIII and Sr-K edgees after selected treatments

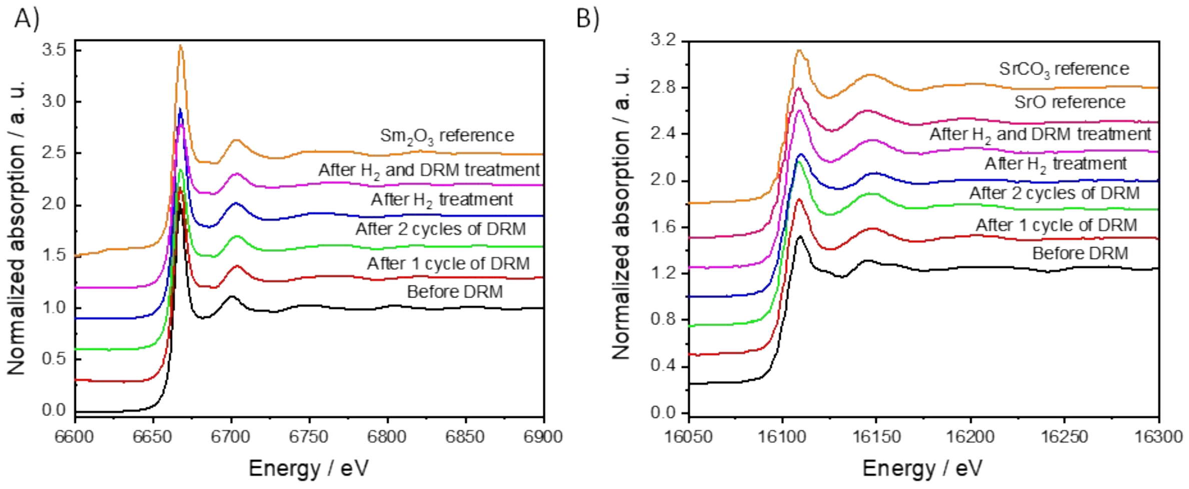

Figure S12: Panel A: Normalized Sm LIII-edge X-ray absorption near-edge structure (XANES) of $Sm_{1.5}Sr_{0.5}NiO_4$ material before and after different DRM experiments. The spectra are compared with that of Sm_2O_3 as a reference material. Panel B: Normalized Sr K-edge X-ray absorption near-edge structure (XANES) of $Sm_{1.5}Sr_{0.5}NiO_4$ material before and after different DRM experiments. The spectra are compared to that of SrO and $SrCO_3$ as a reference material.

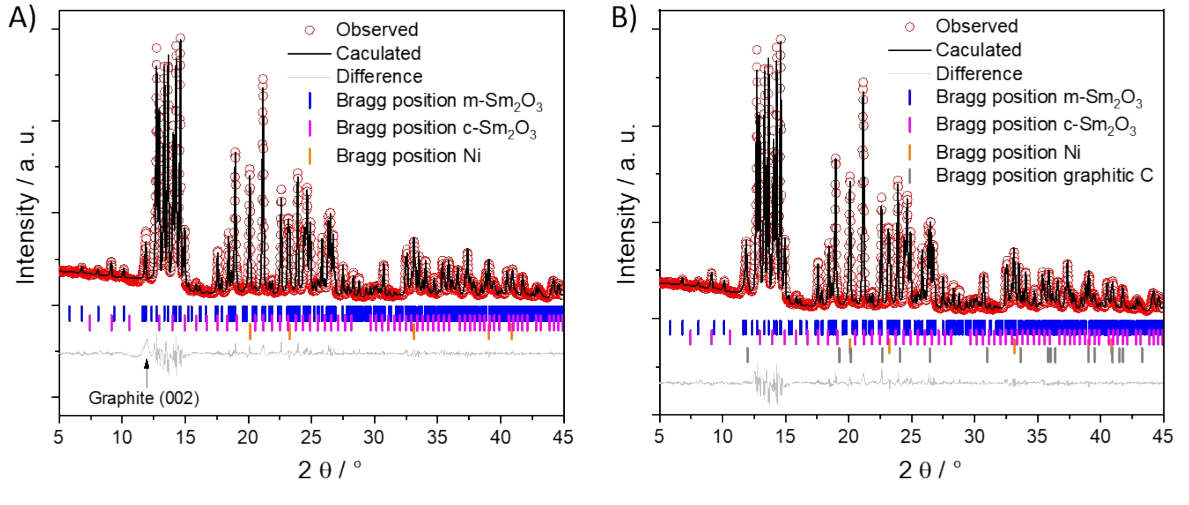

Figure S13: Structure refinement of X-ray powder diffraction data collected at room temperature for Ni/Sm_2O_3 after hydrogen pre-reduction followed by one DRM cycle without (A) and with (B) the inclusion of graphitic carbon in the refinement. The observed (red circle),

calculated (solid black line) and difference (solid gray line) intensities, as well as the calculated Bragg reflections (tick marks) of different crystalline phases. Wavelength: λ=0.7093 Å.

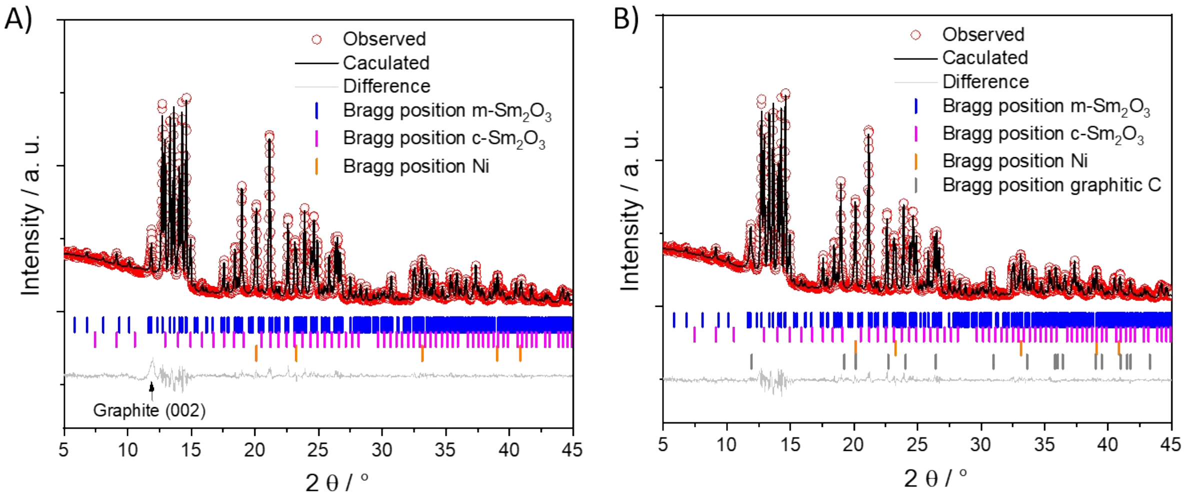

Figure S14: Structure refinement of X-ray powder diffraction data collected at room temperature for Ni/Sm_2O_3 phase after 2 consecutive DRM cycles without (A) and with (B) the inclusion of graphitic carbon in the refinement. The observed (red circle), calculated (solid black line) and difference (solid gray line) intensities, as well as the calculated Bragg reflections (tick marks) of different crystalline phases. Wavelength: λ=0.7093 Å.

Section J: Assessment of Diffusion Limitation

We evaluated the Weisz-Prater criterion for catalysts to prove the absence of diffusion limitation in this work. This criterion is a factor in determining the importance of diffusional limitation:

1. For $\Phi = \frac{r_{obs}L^2}{D_{eff}C_0} < 1$ there is no pore diffusion limitation

2. For $\Phi = \frac{r_{obs}L^2}{D_{eff}C_0} \geq 1$ there is strong pore diffusion limitation,

where r_{obs} is the observed reaction rate in the experiments, L is the characteristic length which is d/6 for spherical particles, D_{eff} is the effective diffusion coefficient, C_0 is the concentration on the catalyst surface. According to the previous descriptions and disregarding the external diffusion resistance (because the catalyst is dispersed with a high void fraction along the catalytic bed and diluted with quartz wool which gives the ability for gas to penetrate easily (concentration on the surface equal to the gas phase), we continue with the calculation.

To calculate D_{eff} [5]: $D_{eff} = \frac{D_{12}\theta}{\tau}$,

where τ is tortuosity factor which is around 2-3 based on the different references [5], θ is the void fraction in the catalyst and D_{12} is the diffusion coefficient of the gases penetrating in porous media along each other.

D_{12} is calculated using the following equation: $D_{12} = \frac{0 \cdot 001858 T^{3/2} [\frac{M_1 + M_2}{M_1 M_2}]^{1/2}}{P \sigma_{12}^2 \Omega_D}$,

where T is the temperature in Kelvin, M is molecular weight of gases, P is pressure which is 1 atm in our study, Ω is the collision integral, a function of kT/ε_{12}, ε and σ are the force constants in the Leonard-Jones potential function, k is the Boltzmann constant =1.38 × 10^{-23} $kgm^2k^{-1}s^{-2}$, $\varepsilon_{12} = \sqrt{\varepsilon_1 \varepsilon_2}$ and $\sigma = \frac{1}{2}(\sigma_1 + \sigma_2)$.

Using the handbooks and references [5] ε and σ for Ar as carrier gas and CH_4 and CO_2 as reactants present in the gas mixture has been gathered and calculations to evaluate D_{eff} and then the Weisz-Prater criterion has been calculated. The results aregathered in the following tables:

Lennard-Jones Force constants					
gas	ε/k k=1.38 × 10^{-23} $kgm^2k^{-1}s^{-2}$	ε	$\varepsilon_{12}=\sqrt{\varepsilon_1 * \varepsilon_2}$	$\sigma(A°)$	$\sigma = \frac{1}{2}(\sigma_1 + \sigma_2)$
Ar	93.3	1.28754 × 10^{-21}		3.542	
			CH_4 in Ar		CH_4 and Ar
CH_4	148.6	2.0507 × 10^{-21}	1.6249 × 10^{-21}	3.758	3.65
			CO_2 in Ar		CO_2 and Ar
CO_2	195.2	2.6937 × 10^{-21}	1.8623 × 10^{-21}	3.941	3.7415

For CH_4 and CO_2 diffusion in Argon through the catalyst, we have calculated the following values based on the above relations and tables in hand books [5], for 3 temperature of studied temperature range are as follows:

T(k)	kT/ε_{12} $CH_{4\ in\ Ar}$	kT/ε_{12} $CO_{2\ in\ Ar}$	Ω CH_4 in Ar	Ω CO_2 in Ar
873	7.41424	6.469097	0.78205	0.80191
973	8.26352	7.210117	0.76708	0.78573

1073	9.1128	7.951136	0.754111	0.77212

According to the mentioned relations and calculated values, further calculations are as follows:

For example, for 1073 K (where the diffusion limitation would be crucial, as at higher temperatures the reaction is faster):

- CH_4 diffusion in Ar inside the catalyst:

D_{12}= ((0.001858*($1073^{1.5}$) *((16+39.948)/ (16*39.948))$^{0.5}$)) / ((3.758^2) * 0.754111)

D_{12}=1.8142 cm^2/s

- CO_2 diffusion in Ar inside the catalyst:

D_{12}= ((0.001858*($1073^{1.5}$) *((44+39.948)/ (44*39.948))$^{0.5}$)) / ((3.941^2) * 0.77212)

D_{12}=1.1901 cm^2/s

With having τ the tortuosity factor which is around 2-3 based on the different references [5] and θ the void fraction of catalyst which is equal to o.4 we can calculate D_{eff}:

CH_4 diffusion in Ar inside the catalyst:

D_{eff}= 0.36284 cm^2/s

CO_2 diffusion in Ar inside the catalyst:

D_{eff}= 0.238 cm^2/s

$D_{eff\ ave}$= 0.3004 cm^2/s

Afterwards one can calculate the $\Phi = \frac{r_{obs}L^2}{D_{eff}C_0}$

According to the results the particle size assuming them as spherical pellets, is below the range of 0.1 mm and at each temperature we have the observed rate which is the change in gas concentration over the time the gas is passing over the catalyst. The C_0 is the concentration on the surface of the catalyst, considering no external diffusion limitation the concentration in the gas phase is the same as the concentration on the catalyst surface.

r_{obs} is also can be calculated according to the proved equations in chemical reactors: $r_{obs}=C_0X_A/t$ in which X is conversion and t is the residence time.

t=V/v , v=100 ml/min or 1.66×10^{-6} m^3/s in our study through a reaction bed with volume of about 10^{-6} m^3. So, t=0.6 s.

For example, at 1073 k, $X_{at\ 1073\ k}$ for catalysts is around 0.9.

After simplifying we result:

$$\Phi = \frac{r_{obs}L^2}{D_{eff}C_0} = \frac{\frac{C_0X_A}{t}L^2}{D_{eff}C_0} = \frac{\frac{X_A}{t}L^2}{D_{eff}}$$

$d_{particle}$ is around 0.01 cm which is clear from the image and has been proved by sieving the powder through different meshes. $L=d_{particle}/6$ is the characteristic length for diffusion inside pores.

$$\Phi = \frac{\frac{X_A \times (\frac{0 \cdot 01\ cm}{6})^2}{0 \cdot 6\ s}}{0.3004\frac{cm^2}{s}}$$

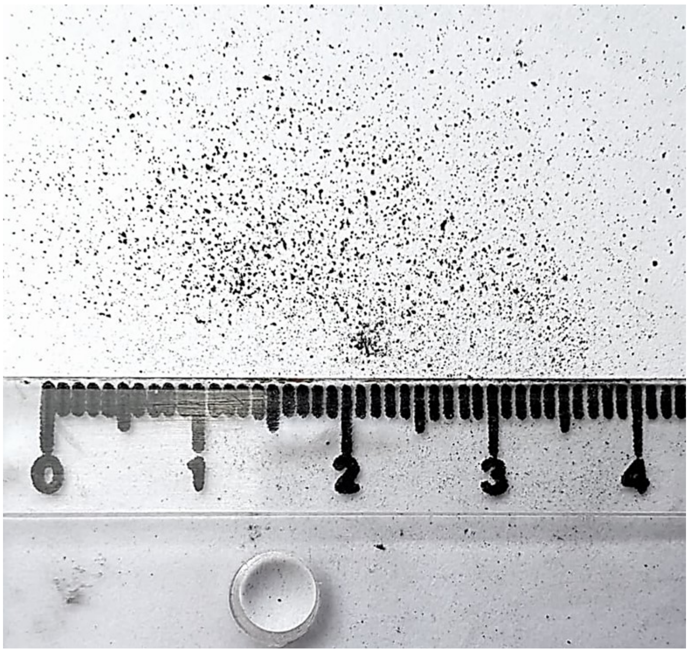

After all calculations at 1073 K: Φ=0.0000138704.

All of the calculated values are well below 1. As all values are Φ<1, there is no diffusion limitation in our catalytic tests. This conclusion proves that for catalysts loaded to the reactor in their powder form and exhibiting small particle sizes, diffusion resistance is not important. The calculations for any other temperatures is in the same value region and still result in Φ<1.

References

[1] O. Levenspiel, *"Chemical Reaction Engineering"*, United States of America, 1999.

[2] Gilbert F. Froment, K.B. Bischoff, *"Chemical Reactor Analysis and Design"*, 1979.

[3] K.B. Bischoff, "An Extension of the General Criterion for Importance of Pore Diffusion with Chemical Reactions", Chemical Engineering Science, 22 (1967) 525-530.

[4] X. Hu, M. Yang, D. Fan, G. Qi, J. Wang, J. Wang, T. Yu, W. Li, M. Shen, *"The Role of Pore Diffusion in Determining NH_3 SCR Active Sites Over Cu/SAPO-34 Catalysts"*, Journal of Catalysis, 341 (2016) 55-61.

[5] C.N. Satterfield, *"Mass Transfer in Heterogeneous Catalysis"*, M.I.T. Press, 1970.
